# Online adaptive group-wise sparse Penalized Recursive Exponentially Weighted N-way Partial Least Square for epidural intracranial BCI

**DOI:** 10.3389/fnhum.2023.1075666

**Published:** 2023-03-06

**Authors:** Alexandre Moly, Alexandre Aksenov, Félix Martel, Tetiana Aksenova

**Affiliations:** ^1^Université Grenoble Alpes, CEA, LETI, Clinatec, Grenoble, France; ^2^Independent Researcher, Montpellier, France

**Keywords:** Brain-Computer Interface, ECoG, penalization, tensor factorization, NPLS, group-wise sparsity, adaptive learning, incremental learning

## Abstract

**Introduction:**

Motor Brain–Computer Interfaces (BCIs) create new communication pathways between the brain and external effectors for patients with severe motor impairments. Control of complex effectors such as robotic arms or exoskeletons is generally based on the real-time decoding of high-resolution neural signals. However, high-dimensional and noisy brain signals pose challenges, such as limitations in the generalization ability of the decoding model and increased computational demands.

**Methods:**

The use of sparse decoders may offer a way to address these challenges. A sparsity-promoting penalization is a common approach to obtaining a sparse solution. BCI features are naturally structured and grouped according to spatial (electrodes), frequency, and temporal dimensions. Applying group-wise sparsity, where the coefficients of a group are set to zero simultaneously, has the potential to decrease computational time and memory usage, as well as simplify data transfer. Additionally, online closed-loop decoder adaptation (CLDA) is known to be an efficient procedure for BCI decoder training, taking into account neuronal feedback. In this study, we propose a new algorithm for online closed-loop training of group-wise sparse multilinear decoders using *L*_*p*_-Penalized Recursive Exponentially Weighted N-way Partial Least Square (PREW-NPLS). Three types of sparsity-promoting penalization were explored using *L*_*p*_ *with p* = 0., 0.5, *and* 1.

**Results:**

The algorithms were tested offline in a pseudo-online manner for features grouped by spatial dimension. A comparison study was conducted using an epidural ECoG dataset recorded from a tetraplegic individual during long-term BCI experiments for controlling a virtual avatar (left/right-hand 3D translation). Novel algorithms showed comparable or better decoding performance than conventional REW-NPLS, which was achieved with sparse models. The proposed algorithms are compatible with real-time CLDA.

**Discussion:**

The proposed algorithm demonstrated good performance while drastically reducing the computational load and the memory consumption. However, the current study is limited to offline computation on data recorded with a single patient, with penalization restricted to the spatial domain only.

## 1. Introduction

Brain–computer interfaces (BCIs) are systems that create a new communication pathway between the brain and an effector without neuromuscular activation. Motor BCIs aim to allow users with severe motor impairments to regain limb mobility by controlling orthoses and prostheses or by recovering motor control over their own limbs, e.g., using electrical stimulation (Mak and Wolpaw, 2009). Most BCI systems include a neural signal acquisition system, a transducer, an effector, and a feedback system (Schwartz et al., 2006). The transducer is typically composed of a neural feature extraction block and a decoder, with optional pre- and post-processing blocks. Decoders are generally user-specific and data-driven. They are created through a supervised tuning of parameters on the training dataset. Once the decoder is established, the transducer can be applied in real time to translate the user's intention into the control command of the effector.

As BCI systems function in real time, computing time and resource management are crucial aspects of such systems (Haufe et al., 2014). High-resolution neuronal activity recording systems are generally required to achieve high-dimensional control of complex effectors. It results in a large volume of data that needs to be processed.

Furthermore, in motor BCI, a high decision rate (8–10 Hz) is necessary to control complex effectors such as robotic arms, an exoskeleton, and so on (Marathe and Taylor, 2015; Shanechi et al., 2017). In addition to high computing power requirements and computing time, the high dimensionality of the feature space presents challenges such as the “curse of dimensionality” during decoder training (Bellman, 1961; Bishop, 2006; Nicolas-Alonso and Gomez-Gil, 2012; Remeseiro and Bolon-Canedo, 2019). The feature space often contains irrelevant and/or redundant features. Moreover, computational load is critical for the potential development of portable BCIs.

Reducing the dimensionality of the neuronal feature space is one approach to addressing these issues. Dimension reduction algorithms have been widely employed in numerous studies on BCI.

Both projection and feature selection methods were applied for dimensional reduction, in both online BCI experiments and offline analysis.

Projections algorithms were often used (Kim et al., 2006; Marathe and Taylor, 2013; Haufe et al., 2014; Bundy et al., 2016; Hsu et al., 2016; Sannelli et al., 2016; Schaeffer and Aksenova, 2016; Eliseyev et al., 2017; Jiang et al., 2017; Seifzadeh et al., 2017; Sreenath and Ramana, 2017; Bousseta et al., 2018; Choi et al., 2018; Lotte et al., 2018; Palmer and Hirata, 2018; Khan et al., 2019; Jafarifarmand and Badamchizadeh, 2020). They project the feature space into a space of a lower dimension by using a linear or non-linear combination of the initial features. The principal and independent component analyses (PCA and ICA), spatio-spectral decomposition (SSD), common spatial pattern (CSP), or partial least squares (PLS) (Kim et al., 2006; Marathe and Taylor, 2013; Haufe et al., 2014; Bundy et al., 2016; Hsu et al., 2016; Sannelli et al., 2016; Schaeffer and Aksenova, 2016; Eliseyev et al., 2017; Jiang et al., 2017; Seifzadeh et al., 2017; Sreenath and Ramana, 2017; Bousseta et al., 2018; Choi et al., 2018; Lotte et al., 2018; Palmer and Hirata, 2018; Khan et al., 2019; Jafarifarmand and Badamchizadeh, 2020) algorithms and variants were applied in BCI research. However, such methods may not improve the computing time as they do not optimize the feature extraction step. The irrelevant and/or redundant features are still computed.

The feature selection family regroups filter-based, wrapper-based, and embedded techniques (Bolón-Canedo et al., 2013; Khaire and Dhanalakshmi, 2019). The filter-based methods rank and select features independently without considering the decoder. Effective in computation time, these methods tend to select highly correlated (redundant) features. The wrapper-based techniques incorporate supervised learning algorithms to evaluate the possible interactions between the features. These techniques add features to the selected subset iteratively and evaluate the subset by combining it with the trained decoder (Lotte et al., 2018). These methods are effective but require a great deal of computing time. On the other hand, embedded techniques integrate the feature selection process directly into the decoding algorithm, thus combining the advantages of both the filter-based and wrapper-based methods (Khaire and Dhanalakshmi, 2019). Embedded feature selection is a promising approach as it is directly performed during the model learning process.

Embedded feature selection in BCI is based on regularization/penalization methods (Cincotti et al., 2008; Lotte and Guan, 2011; Flamary and Rakotomamonjy, 2012; Eliseyev and Aksenova, 2016; Mishra et al., 2018; Nagel and Spüler, 2019). Regularization strategies incorporate a penalty term into the model parameter optimization process to restrict the degree of freedom of the model. Numerous regularization strategies were designed, such as *L*_0_, *L*_1_ (Lasso), *L*_2_ (Ridge), elastic net regularization, and so on. The *L*_1_ regularization process adds a penalty term equal to the sum of the absolute values of model coefficients. *L*_2_ regularization integrates a penalty term equal to the sum of squared values, whereas elastic net regularization is a combination of both *L*_1_ and *L*_2_ penalizations (Bishop, 2006). *L*_0_ (Sreeja and Himanshu, 2019), *L*_1_ (Lotte and Guan, 2011; Eliseyev et al., 2012; Flamary and Rakotomamonjy, 2012; Zhang et al., 2013; López-Larraz et al., 2014), *L*_2_ (Cincotti et al., 2008; Flamary and Rakotomamonjy, 2012; Seifzadeh et al., 2017; Nagel and Spüler, 2019) regularization, and an elastic net (Kim et al., 2018; Peterson et al., 2019) were applied in the BCI field to improve the neural signal decoding performance or other properties, e.g., prediction smoothness or sparsity. Less common regularization methods designed and/or applied in BCI are regularization using polynomial regression (Eliseyev and Aksenova, 2016), sparse regularization based on automatic relevance determination (ARD) (Toda et al., 2011; Nakanishi et al., 2017), and Kullback–Leibler regularization in the Riemannian mean (Mishra et al., 2018). Among regularization techniques, *L*_*p*_ regularization, 0 ≤ *p* ≤ 1, using a penalty equal to the *L*_*p*_ (0 ≤ *p* ≤ 1) norm/pseudo-norm of the model coefficients, is known to be effective in discarding irrelevant/correlated features, promoting a sparse solution (Bishop, 2006; Hastie et al., 2015).

Regularization/penalization is generally performed in a single-wise manner. Features are regularized independently and are not evaluated as belonging to a group of features. However, there are many applications, particularly in BCI, with structurally grouped input features. This approach allows the simultaneous setting of zero of the model coefficients within a group, which can be beneficial in cases where the input features are structurally grouped, such as excluding sensors (Eliseyev et al., 2017). For such applications, a single-wise sparsity-promoting penalty may be suboptimal. Group-wise regularization algorithms perform the feature selection process by grouping and applying penalization to the groups at once (Martínez-Montes et al., 2008; Eliseyev et al., 2012; Giordani and Rocci, 2013; Zhang et al., 2013; Hastie et al., 2015). This grouping can cluster features across various modalities, such as electrodes and frequency bands (van Gerven et al., 2009). Group-wise sparsity is ideal for naturally structured data, allowing for the elimination of variables (such as electrodes or frequency) from the signal processing workflow and reducing computational costs. Additionally, it may simplify the model's interpretation.

Despite being potentially beneficial for studies on BCI, group-wise penalization has rarely been applied in this field. The natural structure of BCI features space grouping features over modalities, such as electrodes, frequency bands, and time delay, which are heavily exploited (van Gerven et al., 2009; Eliseyev et al., 2012; Motrenko and Strijov, 2018; Wu et al., 2019). For the penalization of such groups, data may be viewed in the form of a tensor (Hastie et al., 2015; Eliseyev and Aksenova, 2016). Tensors, or multiway arrays, are higher-order generalizations of vectors and matrices (for more details, see Ref. Hsu et al., 2016). Tensors have several dimensions, also known as ways of analysis or modes. In BCIs, recorded signals are primarily analyzed in spatial, frequency, and temporal domains. To extract neural features, each epoch of neural signal recording is commonly mapped to temporal-frequency-spatial (or frequency-spatial) space (e.g., Chao et al., 2010; Schaeffer and Aksenova, 2016; Eliseyev et al., 2017; Choi et al., 2018). Tensor-based analyses, which present features in matrix form using tensor unfolding, are reported to be beneficial for BCI decoding as they preserve the natural structure of data (Zhao et al., 2013; Cichocki et al., 2015; Zhang et al., 2015; Eliseyev et al., 2017).

The tensors of data in BCI often benefit from tensor decomposition techniques. Slice-wise data representation can be obtained through sparsity-promoting penalization of tensor decomposition. Regularized PARAFAC and Tucker decomposition are algorithms designed for group-wise tensor penalization. Slice-wise sparsity-promoting penalization is added to the N-way Partial Least Square (NPLS) in van Gerven et al. (2009), Eliseyev et al. (2012), Motrenko and Strijov (2018), and Wu et al. (2019). These techniques have been used in a few offline BCI studies (Martínez-Montes et al., 2008; Eliseyev et al., 2012) and in other fields (Giordani and Rocci, 2013; Kim et al., 2013, 2014; Hervás et al., 2019). In Eliseyev et al. (2012), the *L*_1_-Regularized N-PLS algorithm was shown to be superior to its non-penalized version by suppressing noisy/irrelevant electrodes. However, these algorithms were not adapted for online decoder training in closed-loop use.

Most of the presented feature-dimensional reduction algorithms were tested offline. Additionally, feature selection performed in an offline preliminary study (Brunner et al., 2006; Huang et al., 2009; Spüler et al., 2012; Marathe and Taylor, 2013; Bousseta et al., 2018; Cantillo-Negrete et al., 2018; Kim et al., 2018; Nagel and Spüler, 2019) may not be optimal when using a CLDA strategy (Schlögl et al., 2010; Clerc et al., 2016). CLDA involves training the decoder online on data acquired during closed-loop BCI control sessions. Decoders trained in this manner have been reported to outperform decoders trained offline using data from open-loop BCI experiments (Jarosiewicz et al., 2013). Adaptive/incremental learning algorithms are particularly suited for the CLDA strategy. These algorithms continuously update the model, using only the latest data block and relevant statistics on the older signals, while not retaining the whole signals in memory (Schlögl et al., 2010; Brandman et al., 2018; Lotte et al., 2018). Adaptive/incremental learning algorithms are beneficial for the CLDA training strategy as they allow higher decoder update rates and are compatible with long decoder learning periods, which are generally necessary for high-dimensional control. However, only a few adaptive dimensional reduction algorithms were proposed. The adaptive dimensional reduction algorithms applied in the BCI (Zhao et al., 2008; Ang et al., 2011; Song and Yoon, 2015; Woehrle et al., 2015; Hsu et al., 2016; Mobaien and Boostani, 2016; Sannelli et al., 2016; Chen and Fang, 2017; Lotte et al., 2018) and other (Dagher, 2010) fields are primarily based on projection strategies (adaptive CSP, PCA, ICA, and xDAWN algorithms) and were only tested offline. Similarly, a few adaptive feature selection algorithms were proposed. Filter methods were tested on BCI simulations using mutual information (Oliver et al., 2013) or during online BCI experiments based on the Fisher score (Faller et al., 2012). The wapper-based strategy was optimized using parallel computation for the online BCI classifier (Mend and Kullmann, 2012), whereas embedded methods using semi-supervised feature selection (Long et al., 2011) and a weighting feature algorithm (Andreu-Perez et al., 2018) have been designed and used during online BCI applications. An adaptive genetic algorithm was proposed for adaptive channel selection in Moro et al. (2017). However, these methods have been applied to simple online binary classification only.

In BCI studies, adaptive regularization algorithms were tested offline (Roijendijk et al., 2016; Mishra et al., 2018; Sharghian et al., 2019). Adaptive algorithms with an *L*_1_ norm regularization strategy were reported in an adaptive logistic regression (Sheikhattar et al., 2015), a kernel least squares (Yang et al., 2019), and recursive least squares algorithms (Chen et al., 2012) in other fields. Only a few feature selection methods were integrated into adaptive algorithms for online incremental calibration during real-time BCI experiments and were generally restricted to binary classification and EEG-based experiments (Long et al., 2011; Faller et al., 2012; Mend and Kullmann, 2012; Moro et al., 2017; Andreu-Perez et al., 2018). Computational complexity and difficulty integrating dimensional reduction methods into real-time algorithms may explain the lack of proposed solutions.

In the study, an adaptive algorithm promoting group-wise model sparsity, *L*_*p*_-penalized recursive exponentially weighted N-way Partial Least Square (PREW-NPLS), is proposed. *L*_*p*_, *p* = 0, 0.5, 1 norm/pseudo-norm penalty is applied to feature groups corresponding to the slices of data tensor related to the mode of analysis (e.g., spatial, frequency, temporal). The algorithm was tested with data recorded during BCI sessions of left/right arm 3D translations of a virtual avatar by a tetraplegic patient during the clinical trial NCT02550522 (ClinicalTrials.gov) conducted at the Grenoble Alpes University Hospital (CHUGA) (Benabid et al., 2019; Moly et al., 2022). The datasets were recorded during online closed-loop experiments using the REW-NPLS decoder that was previously integrated into the BCI system. To reproduce online experimental conditions and to ensure that the proposed algorithm is compatible with real-time CLDA, a pseudo-online simulation was conducted using the same parameters (buffer size, batch training, and so on) and the same model application procedure as it was used in real time. The comparison study was restricted to penalization according to spatial modality, which is the most critical at BCIs due to data recording/transfer limitations. For each type of penalization, a set of models/penalization parameters were evaluated. The PREW-NPLS decoders highlighted equivalent or better decoding performance compared to the generic REW-NPLS algorithm for the majority of the penalization parameters. The sparsest solutions allowed the removal of up to 75% of the electrodes without decreasing performance. *L*_0_-PREW-NPLS and *L*_1_-PREW-NPLS are sufficiently computationally efficient for online closed-loop decoder adaptation at a high-frequency rate.

## 2. Methods

### 2.1. Generic partial least squares and family

Algorithms of the PLS family are widely used in BCI studies due to their stability in the case of high-dimensional data and in the presence of correlated and/or irrelevant variables. In motor BCI, the algorithms of the PLS family were applied in both continuous and discrete BCIs. Offline hand/fingers trajectory decoding (Chao et al., 2010; Chen et al., 2013; Eliseyev and Aksenova, 2014; Bundy et al., 2016; Schaeffer and Aksenova, 2016; Schaeffer, 2017; Choi et al., 2018), real-time hand translation/wrist rotation control (Benabid et al., 2019; Moly et al., 2022), error potential (ERP) detection (Rouanne et al., 2021) from ECoG, and EEG/MEG-based classification (Trejo et al., 2006; Eliseyev et al., 2017; Maleki et al., 2018) using the PLS algorithm were reported in preclinical and clinical studies. In Eliseyev and Aksenova (2014), GAM-PLS (generalized additive model—the partial least square) is reported to outperform the generic PLS in the presence of artifacts for 3D hand trajectory decoding from ECoG data in non-human primates (NHP). In Schaeffer (2017), PLS outperformed principle component regression and demonstrated comparable results with Lasso regression for hand trajectory and 1D finger trajectory decoding from ECoG in preclinical and clinical experiments. In Rouanne et al. (2021), NPLS demonstrated comparable results with Logistic Regression, SVM, MLP, and CNN for ERP detection from ECoG recordings in the sensory-motor cortex of tetraplegics, etc.

The generic PLS is a linear regression algorithm based on the iterative projection of input and output variables into the latent variable spaces of dimension *f*. The hyperparameter *f* is generally estimated through cross-validation in the preliminary study. Projectors are set to maximize the covariance between the input and latent output variables. The generic PLS is an offline algorithm. For online data stream modeling, recursive PLS (RPLS) and recursive exponentially weighted PLS (REW PLS) (Helland et al., 1992; Dayal and MacGregor, 1997; Qin, 1998) were developed. All the aforementioned PLS algorithms are vector-input-vector-output algorithms. N-way Partial Least Square (NPLS) is a generalization of the conventional PLS for tensor data (Bro, 1996, 1998). The NPLS algorithm projects the input and output tensors into a low-dimensional space of latent variables using a low-rank tensor decomposition. The recursive N-way PLS (RNPLS) (Helland et al., 1992) and recursive exponentially weighted N-way PLS (REW-NPLS) (Dayal and MacGregor, 1997) are generalizations of the adaptive RPLS and REW PLS algorithms to tensor variables and allow online tensor data stream learning of the regression model. RNPLS still requires fixing the hyperparameter *f* from the offline preliminary study, whereas REW-NPLS proposes a recursive validation procedure for the online optimization of the hyperparameter, enabling a fully adaptive algorithm (Dayal and MacGregor, 1997). In addition, the REW-NPLS algorithm is more computationally effective than the RPLS algorithm (Dayal and MacGregor, 1997).

The adaptive REW-NPLS algorithm has been tested offline in BCI studies for trajectory decoding from ECoG signals and for classification from MEG data, demonstrating similar or better results compared to other algorithms from the PLS family designed for offline use (Eliseyev et al., 2017). It was applied in real time for closed-loop adaptation of 3D hand translation/wrist rotation decoders in tetraplegics (Benabid et al., 2019; Moly et al., 2022). Finally, REW-NPLS was tested in the simulation of auto-adaptive continuous (bi-directional cursor control) and discrete multiclass motor imagery (MI) BCI in a tetraplegic patient (Rouanne et al., 2022). In the (Sliwowski et al., 2022) offline study, ANN algorithms were reported to outperform the REW-NPLS decoder. However, these algorithms cannot be applied in real time under CLDA.

Fully adaptive REW-NPLS is compatible with CLDA. However, it may be further improved by integrating real-time adaptive dimension reduction and promoting group-wise decoder sparsity using regularization. Group-wise sparsity, e.g., in the spatial dimension, may allow the elimination of irrelevant or highly correlated electrodes, decreasing computational time and memory consumption at the BCI use stage. This may be critical for portable BCI systems. A sparse solution may be advantageous for small training data sets, preventing overtraining. As BCI decoders require regular updates due to neuronal signal non-stationarity, reducing the decoder training time is desirable for real-life scenarios.

### 2.2. REW-NPLS

NPLS (Bro, 1996, 1998) estimates a linear relationship between a tensor of independent (input) and a tensor of dependent (output) variables. Given **X**_*t*_ $\in \;{\mathbb{R}}^{I_{1}\times \ldots \times I_{m}}$ and **Y**_*t*_$\in {\mathbb{R}}^{J_{1}\times \ldots \times J_{n}}$ the *m* and *n* order tensors of the input and output variables at time *t*, **Y**_*t*_ = **Beta**_*t*_+**bias**+**D**_*t*_, where **Beta** and **bias** are the tensors of parameters and their associated bias, ${\underline{D}}_{t}\in {\mathbb{R}}^{J_{1}\times \ldots \times J_{n}}$ is the tensor of noise. The parameters are estimated from the training dataset $\left\{\underline{X},\underline{Y}\right\}$, $\underline{X}\in {\mathbb{R}}^{L\times I_{1}\times \ldots \times I_{m}}$, $\underline{Y}\in {\mathbb{R}}^{L\times J_{1}\times \ldots \times J_{n}}$, *L* is the training dataset size. NPLS constructs the linear regression iteratively by projecting tensors of observation $\underline{X}$ and $\underline{Y}$ to the space of latent variables using tensor decomposition: $\underline{X}=\sum_{f_{i}=1}^{f}r_{f_{i}}\circ \;w_{f_{i}}^{1}\;\circ \ldots \circ \;w_{f_{i}}^{m}+{\underline{E}}_{x}$, $\underline{Y}=\sum_{f_{i}=1}^{f}u_{f_{i}}\circ \;q_{f_{i}}^{1}\circ \ldots \circ \;q_{f_{i}}^{n}+{\underline{E}}_{y}$. Here, “∘” is the outer product operator (Eliseyev et al., 2017), ${\text{R}}_{f}=\left[{\text{r}}_{1},\ldots ,{\text{r}}_{f}\right]\in {\mathbb{R}}^{L\times f}$ and ${\text{U}}_{f}=\left[{\text{u}}_{1},\ldots ,{\text{u}}_{f}\right]\in {\mathbb{R}}^{L\times f}$ are matrices of the latent variables, $W^{f_{i}}={\left\{w_{f_{i}}^{i}\right\}}_{i=1}^{m}$, ${\text{w}}_{f_{i}}^{i}\in {\mathbb{R}}^{I_{i}}$ and $Q^{f_{i}}={\left\{{\text{q}}_{f_{i}}^{i}\right\}}_{i=1}^{n}$, ${\text{q}}_{f_{i}}^{i}\in {\mathbb{R}}^{J_{i}}$, *f*_*i*_ = 1, …, *f* are the projection vectors constructed at iteration *f*_*i*_, and **E**_*x*_, **E**_*y*_ are the residual tensors. The set of projectors is constructed iteratively, increasing latent variable space dimensions.

REW-NPLS (Eliseyev et al., 2017) update a set of *F* (*F* ∈ ℕ^*^ is the fixed upper bound latent space dimension) models ${\left\{{\underline{Beta}}_{UI}^{f},{\underline{bias}}_{UI}^{f}\right\}}_{f=1}^{F}$ using current blocks of tensors of observation {**X**_*UI*_, **Y**_*UI*_} and previously computed models. Here, *UI* ∈ ℕ is the updated iteration number, $\text{ ​​}\;\text{​}{\underline{Beta}}_{UI}^{f}\in {\mathbb{R}}^{\left(I_{1}\times \ldots \times I_{M})\times (J_{1}\times \ldots \times J_{N}\right)}$, $\text{ ​​}\;\text{​​ }{\underline{bias}}_{UI}^{f}\in {\mathbb{R}}^{J_{1}\times \ldots \times J_{N}}$ are current update of model coefficients, and ${\underline{X}}_{UI}\in {\mathbb{R}}^{\text{Δ}L\times I_{1}\times \ldots \times I_{M}}$, ${\underline{Y}}_{UI}\in {\mathbb{R}}^{\text{Δ}L\times J_{1}\times \ldots \times J_{N}}$ are the current input and output tensors of observations. Δ*L* ∈ ^*^ is the number of samples recorded between the update blocks *UI* − 1 and *UI*.

The REW-NPLS algorithm is a generalization of the REW-PLS algorithm to tensor variables and belongs to the family of kernel PLS algorithms (Eliseyev et al., 2017). It evaluates a set of projectors and model coefficients from covariance tensors $\underline{XY}=\underline{X}\;\times_{1}\underline{Y}$, $\underline{XY}\in {\mathbb{R}}^{\left(I_{1}\times \ldots \times I_{m}\right)\times \left(J_{1}\times \ldots \times J_{n}\right)}$, and $\underline{XX}=\underline{X}\times_{1}\underline{X}$, $\underline{XX}\in {\mathbb{R}}^{\left(I_{1}\times \ldots \times I_{m})\times (I_{1}\times \ldots \times I_{m}\right)}$. Here, “ × _*k*_” is the k-mode tensor product (Eliseyev et al., 2017). First, a set of input variable projectors *W* are evaluated from the covariance tensor **XY**. The projectors are estimated using a rank one decomposition of the tensor $\underline{V}=\;\underline{XY}\in {\mathbb{R}}^{I_{1}\times \ldots \times I_{m}}$ in the case of single output. For higher dimensions, the eigenvector with the largest eigenvalue is computed from the covariance tensor **XY** to decrease the dimension of the tensor to decompose: **e** = *eig*(**XY**^*T*^**XY**), $\underline{V}=reshape\;(XY\cdot e),\underline{V}\in^{I_{1}\times \ldots \times I_{m}}$. Here, $XY\in {\mathbb{R}}^{\left(I_{1}\cdot \ldots \cdot I_{m})\times (J_{1}\cdot \ldots \cdot J_{n}\right)}$ is the unfolded tensor **XY**, and **e** = *eig*() is an eigenvector with the largest eigenvalue. Output projectors and the model parameters **Beta** and **bias** are computed using *W* and covariance tensors **XX** and **XY**. The projectors sets and model parameters are evaluated sequentially, increasing the latent variables' space dimension in the internal REW-NPLS iterations *f*.

Finally, at the *UI*th update, the covariance tensors **XX**_*UI*_ and **XY**_*UI*_ are computed from the previous **XX**_*UI*−1_ and **XY**_*UI*−1_ tensors, and the current block of observations {**X**_*UI*_, **Y**_*UI*_}:


$${\underline{XX}}_{UI}=\mu_{1}{\underline{XX}}_{UI-1}+{\underline{X}}_{UI}\times_{1}{\underline{X}}_{UI},$$

$${\underline{XY}}_{UI}=\mu_{1}{\underline{XY}}_{UI-1}+{\underline{X}}_{UI}\times_{1}{\underline{Y}}_{UI}$$

μ_1_ is a forgetting factor. A set of projectors is evaluated using a rank-one tensor decomposition (Eliseyev et al., 2017) of the current tensor *V*_*UI*_.

In the REW-NPLS algorithm, only the covariance tensors, the normalization coefficients, and the current model are stored together with the current block of observations collected since the previous update.

### 2.3. PARAFAC procedure in REW-NPLS

Several tensor decomposition strategies were designed: the Parallel factor analysis (PARAFAC), Tucker, multilinear SVD decomposition, and so forth (Cichocki et al., 2015). Similar to generic NPLS, the REW-NPLS algorithm employs PARAFAC tensor decomposition (Eliseyev et al., 2017). PARAFAC or CANDECOMP/PARAFAC (CP), also known as polyadic decomposition (PD), can be considered the generalization of principal component analysis (PCA) and singular value decomposition (SVD) to the tensor case (Sheikhattar et al., 2015; Sharghian et al., 2019). This method represents a *M*-order tensor $\underline{V}\in {\mathbb{R}}^{I_{1}\times \ldots \times I_{m}}$ as the linear combination of vectors' outer products (rank-one tensors) such as follows:


$$\underline{V}=\sum_{r=1}^{R}\rho_{r}w_{r}^{1}\circ w_{r}^{2}\circ \ldots \circ w_{r}^{m}+\underline{E},$$

$$\left\|w_{r}^{i}\right\|=1,\text{​​ ​​ }r=1,\ldots ,R;\text{​​ ​​ }i=1,\ldots ,m.$$

Here, 1 ≤ *i* ≤ *m* corresponds to the *i*^*th*^ mode/dimension of the tensor variable, “∘” is the (vector) outer product of the decomposition factors (projectors) ${\text{w}}_{r}^{i}\in {\mathbb{R}}^{I_{i}}$, *R* ∈ ℕ is the number of rank-one tensors used for decomposition, ρ_*r*_ is the weight associated with each rank-one tensor of the decomposition and $\underline{E}\in {\mathbb{R}}^{I_{1}\times \ldots \times I_{m}}$ is the residual tensor (Kolda and Bader, 2009). PARAFAC evaluates the projectors, minimizing the residuals.

Similar to generic NPLS, only one step of PARAFAC (*R* = 1) is applied to the current tensor decomposition at each internal iteration *f*_*i*_ = 1, …, *f* of REW-NPLS:


$$\underline{V}=\rho_{r}w^{1}\circ w^{2}\circ \ldots \circ w^{m}+\underline{E},$$

$$\left\|w^{1}\right\|=1,\text{​​ ​​ }i=1,\ldots ,m.$$

To solve the optimization problem, the alternating least squares (ALS) algorithm is employed in REW-NPLS. ALS optimizes one projector iteratively at a time and fixes others, reducing, at each iteration of ALS, the optimization problem to a least-squares linear regression (Kolda and Bader, 2009; Cichocki et al., 2015; Pereira Da Silva et al., 2015).

REW-NPLS includes several iterations inside other iterative procedures: ALS iterations for PARAFAC (*R* = 1) decomposition, internal REW-NPLS iterations increasing latent variables space dimension, and, finally, the update iterations *UI*.

### 2.4. *L*_*p*_-Penalized REW-NPLS (PREW-NPLS)

Sparse input variables projectors may result in a sparse model. Variable excluded from all the projectors $W^{f_{i}}$, *f*_*i*_ = 1, …, *f* is excluded from consideration. As for the tensor data, the projection is made according to the mode of analysis (e.g., special, frequency, or temporal). The sparsity of the projectors may allow excluding the slices of data from the model (e.g., exclude non-informative or redundant electrodes). To achieve sparse projectors, the proposed PREW-NPLS algorithm employed a penalized version of the PARAFAC (*R* = 1), introducing sparsity-promoting penalization. *L*_*p*_, *p* = 0, 0.5, 1 penalization, being the classic lasso regularization (*L*_1_) or less conventional *L*_0_ and *L*_0.5_ penalization is studied. This section describes *L*_*p*_-Penalization in PARAFAC (*R* = 1) and its integration into the REW-NPLS algorithm to build a new sparsity-promoting adaptive PREW-NPLS algorithm.

#### 2.4.1. *L*_*p*_-penalized PARAFAC (*R* = 1)

To simplify the notations without losing generality, a case of a three-order tensor is considered in the section. Given a three-order tensor, one step of PARAFAC (*R* = 1) applied at each iteration of REW-NPLS solves the optimization problem:


(1) $$\underset{w^{1},w^{2},w^{3}}{\min}\|\underline{V}-\rho w^{1}\circ w^{2}\circ w^{3}{\|}^{2},$$

$$\left\|w^{1}\right\|=\left\|w^{2}\right\|=\left\|w^{3}\right\|=1,$$

$\underline{V}\in {\mathbb{R}}^{I_{1}\times I_{2}\times I_{3}}$ and $w^{i}\in {{\mathbb{R}}^{*}}^{I_{i}}$, *i* = 1, 2, 3. As the norms of the projectors are arbitrary values in (1), the decomposition vectors are evaluated, minimizing $\underline{V}-w^{1}\;\circ \;w^{2}\;\circ \;w^{3}{}^{{}^{2}}$ before being normalized. The optimization problem (1) is solved using the ALS procedure. At each step, ALS fixes two of the three vectors **w**^1^, **w**^2^, **w**^3^ reducing the problem to a linear least-squares optimization, e.g. **w**^2^, **w**^3^ are fixes to approximate **w**^1^ and then **w**^2^ is evaluated fixing **w**^1^, **w**^3^ etc. until convergence (Uschmajew, 2015):


$$\underset{w^{1}}{\min}{\left\|{\underset{-}{V}}_{\left(1\right)}-w^{1}{\left(w^{3}⊗w^{2}\right)}^{\text{T}}\right\|}^{2},$$

$$\underset{w^{2}}{\min}{\left\|{\underset{-}{V}}_{\left(2\right)}-w^{2}{\left(w^{3}⊗w^{1}\right)}^{\text{T}}\right\|}^{2},$$

$$\underset{w^{3}}{\min}{\left\|{\underset{-}{V}}_{\left(3\right)}-w^{3}{\left(w^{2}⊗w^{1}\right)}^{\text{T}}\right\|}^{2}.$$

Here, ${\underline{V}}_{\left(i\right)}=\left(v_{1}^{1}\ldots v_{1}^{I_{1}}\right)\in R^{I_{1}\times I_{2}I_{3}}$ is the tensor $\underline{V}$ unfolded according to *i*-the direction and ⊗ is the Kronecker product. Taking into account that ${\left(w^{2}⊗w^{1}\right)}^{\text{T}}\in R^{I_{1}I_{2}},\;{\left(w^{3}⊗w^{1}\right)}^{\text{T}}\in R^{I_{1}I_{3}}$ and ${\left(w^{3}⊗w^{2}\right)}^{\text{T}}\in R^{I_{2}I_{3}}$ are vectors, the optimization tasks are separated into element-wise optimizations:


(2) $$\underset{{\text{w}}_{j}^{1}}{\min}{\left\|v_{1}^{j}-{\text{w}}_{j}^{1}{\left(w^{3}⊗w^{2}\right)}^{\text{T}}\right\|}^{2}\;j=1,\ldots ,I_{1},$$

(3) $$\underset{{\text{w}}_{j}^{2}}{\min}{\left\|v_{2}^{j}-{\text{w}}_{j}^{2}{\left(w^{3}⊗w^{1}\right)}^{\text{T}}\right\|}^{2}\;j=1,\ldots ,I_{2},$$

(4) $$\underset{{\text{w}}_{j}^{3}}{\min}{\left\|v_{3}^{j}-{\text{w}}_{j}^{3}{\left(w^{2}⊗w^{1}\right)}^{\text{T}}\right\|}^{2}\;j=1,\ldots ,I_{3}.$$

Here, $w^{1}={\left({\text{w}}_{1}^{1},\ldots ,\;{\text{w}}_{I_{1}}^{1}\right)}^{T}\in {{\mathbb{R}}^{\text{*}}}^{I_{1}}$, $w^{2}={\left({\text{w}}_{1}^{2},\ldots ,\;{\text{w}}_{I_{2}}^{2}\right)}^{T}\in {{\mathbb{R}}^{\text{*}}}^{I_{2}}$, and $w^{3}={\left({\text{w}}_{1}^{3},\ldots ,\;{\text{w}}_{I_{3}}^{3}\right)}^{T}\in {{\mathbb{R}}^{\text{*}}}^{I_{3}}$. The least square (LS) solutions of (2)–(4) are:


(5) $${\left({\text{w}}_{j}^{1}\right)}_{LS}=\frac{v_{1}^{j}\left(w^{3}⊗w^{2}\right)}{{\left\|w^{3}⊗w^{2}\right\|}^{2}},\;j=1,\ldots ,I_{1},$$

(6) $${\left({\text{w}}_{j}^{2}\right)}_{LS}=\frac{v_{2}^{j}\left(w^{3}⊗w^{1}\right)}{{\left\|w^{3}⊗w^{1}\right\|}^{2}},\;j=1,\ldots ,I_{2},$$

(7) $${\left({\text{w}}_{j}^{3}\right)}_{LS}=\frac{v_{3}^{j}\left(w^{2}⊗w^{1}\right)}{{\left\|w^{2}⊗w^{1}\right\|}^{2}},\;j=1,\ldots ,I_{3}.$$

In this study, sparse promoting penalization using *L*_*p*_, *p* = 0, 0.5, 1 norm/pseudo norm is proposed to be integrated into the cost function of PARAFAC (*R* = 1) to provide a slice-wise sparsity to the solution. The optimization task (1) is replaced by:


(8) $${\left\|\underset{-}{V}-\hat{\underline{V}}\right\|}^{2}+\text{P}\left(w^{1},w^{2},w^{3}\right)\to min,$$

$$\underline{\hat{V}}=\rho w^{1}\circ w^{2}\circ w^{3},$$

$$\text{P}\left(w^{1},w^{2},w^{3}\right)=\lambda_{1}{\left\|w^{1}\right\|}_{q,L_{1}}+\lambda_{2}{\left\|w^{2}\right\|}_{q,L_{2}}+\lambda_{3}{\left\|w^{3}\right\|}_{q,L_{3}},$$

$$\left\|w^{1}\right\|=\left\|w^{2}\right\|=\left\|w^{3}\right\|=1.$$

Here, ${\left\|w^{i}\right\|}_{p,{ℒ}_{i}}$ for *p* = 0 , 0.5, 1 and *i* = 1, 2, 3 is denoted as


$${\left\|w^{i}\right\|}_{0,L_{i}}=\sum_{k\in L_{i}}\left(1-\delta_{0,{\text{w}}_{k}^{i}}\right),$$

$${\left\|w^{i}\right\|}_{1,L_{i}}=\sum_{k\in L_{i}}\left|{\text{w}}_{k}^{i}\right|\;,$$

and


$${\left\|w^{i}\right\|}_{\frac{1}{2},L_{i}}=\sum_{k\in L_{i}}\sqrt{\left|{\text{w}}_{k}^{i}\right|}.$$

0 < λ_*i*_ ≤ 1 are regularization coefficients, the Kronecker delta $\delta_{0,{\text{w}}_{k}^{i}}=1$ if ${\text{w}}_{k}^{i}=0$, and $\delta_{0,{\text{w}}_{k}^{i}}=0$ otherwise. Notably, only a part of the indices defined by a set ${ℒ}_{i}\;\subset \left\{1,2,\ldots ,I_{i}\right\}$, *i* = 1, 2, 3 are penalized, resulting in selective penalization (Lutay and Khusainov, 2021). A set ${ℒ}_{i}\;$ may vary depending on the REW-NPLS iteration. Selective penalization is introduced for the integration of *L*_*p*_. -Penalized PARAFAC (*R* = 1) into the REW-NPLS algorithm.

Similar sparse promoting penalization in PARAFAC (*R* = 1) was considered in Eliseyev et al. (2012). However, this study was limited to *L*_1_-norm penalization. Integrated with the conventional non-adaptive NPLS algorithm, the optimization problem was solved using time consuming numerical optimization, which was then applied offline. In the current manuscript, a more general case of *L*_*p*_, *p* = 0, 0.5, 1 norm/pseudo norm penalization is considered. In addition, selective penalization is applied for efficient integration of penalized PARAFAC into the REW-NPLS algorithm. Finally, an efficient optimization procedure compatible with real-time online applications is proposed.

The same ALS strategy is applied to complete the optimization task (8). ALS fixed all projectors at each step except one, leading to three successive optimization tasks. The least square (LS) sotion $w_{LS}^{i}$ of the non-regularized problem is used as an initial approximation. Notably, unlike in non-regularized optimization, due to the penalization terms, the norms of the projectors are not arbitrary values anymore. Therefore, the normalization of a current estimate is added to ALS optimization iterations:


(9) $$\underset{{\tilde{w}}^{1}}{\min}\left({\left\|{\underset{-}{V}}_{\left(1\right)}-{\tilde{w}}^{1}{\left(w^{3}⊗w^{2}\right)}^{\text{T}}\right\|}^{2}+\lambda_{1}{\left\|{\tilde{w}}^{1}\right\|}_{q,L_{1}}\right),\;w^{1}={\tilde{w}}^{1}/\left\|{\tilde{w}}^{1}\right\|,$$

(10) $$\underset{{\tilde{w}}^{2}}{\text{min}}\left({\left\|{\underset{-}{V}}_{\left(2\right)}-{\tilde{w}}^{2}{\left(w^{3}⊗w^{1}\right)}^{\text{T}}\right\|}^{2}\text{​}+\text{​}\lambda_{2}{\left\|{\tilde{w}}^{2}\right\|}_{q,L_{2}}\right),\text{​}\;w^{2}\text{​}=\text{​}{\tilde{w}}^{2}/\left\|{\tilde{w}}^{2}\right\|,$$

(11) $$\underset{{\tilde{w}}^{3}}{\text{min}}\left({\left\|{\underset{-}{V}}_{\left(3\right)}-{\tilde{w}}^{3}{\left(w^{2}⊗w^{1}\right)}^{\text{T}}\right\|}^{2}\text{​}+\text{​}\lambda_{3}{\left\|{\tilde{w}}^{3}\right\|}_{q,L_{3}}\right),\text{​}\;w^{3}\text{​}=\text{​}{\tilde{w}}^{3}/\left\|{\tilde{w}}^{3}\right\|.$$

It should be noted that all considered regularization functions are decomposed as a sum of element-wise functions. Consequently, similarly to (3)–(5) optimization tasks, (9)–(11) are split into element-wise optimizations:


(12) $$\underset{{\text{w}}_{j}^{1}}{\text{min ​​}\;\text{​​ }}\left({\left\|v_{1}^{j}-{\text{w}}_{j}^{1}{\left(w^{3}⊗w^{2}\right)}^{\text{T}}\right\|}^{2}+\lambda_{1}\times g_{p}({\text{w}}_{j}^{1})\right),\;j=1,\text{​​}\ldots ,\;I_{1},$$

(13) $$\underset{{\text{w}}_{j}^{2}}{\text{min ​​}\;\text{​​ }}\left({\left\|v_{2}^{j}\text{​}-{\text{w}}_{j}^{2}{\left(w^{3}⊗w^{1}\right)}^{\text{T}}\right\|}^{2}\text{​}+\text{​}\lambda_{2}\times g_{p}\left({\text{w}}_{j}^{2}\right)\text{​}\right),\;j=1,\text{​}\ldots ,\text{​}I_{2},$$

(14) $$\underset{{\text{w}}_{j}^{3}}{\text{min ​​}\;\text{​​ }}\left({\left\|v_{3}^{j}-{\text{w}}_{j}^{3}{\left(w^{2}⊗w^{1}\right)}^{\text{T}}\right\|}^{2}\text{​}+\lambda_{3}\times g_{p}({\text{w}}_{j}^{3})\right),\;j=1,\text{​}\ldots ,\text{​}I_{3},$$

(15) $$g_{p}({\text{w}}_{j}^{i})=\{\begin{array}{c} \begin{array}{c} \begin{array}{c} \begin{array}{cc} 1-\delta_{0,{\text{w}}_{j}^{i}},\; & \text{ ​​ ​​ if }p=0\text{ and }{\text{w}}_{j}^{i}\in {ℒ}_{i} \end{array}\\ \begin{array}{cc} \left|{\text{w}}_{j}^{i}\right|, & \text{if }p=1\text{ and }{\text{w}}_{j}^{i}\in {ℒ}_{i}\; \end{array} \end{array}\\ \begin{array}{cc} \sqrt{\left|{\text{w}}_{j}^{i}\right|}\text{ ​​ ​​ }, & \text{if }p=1/2\text{ and }{\text{w}}_{j}^{i}\in {ℒ}_{i} \end{array} \end{array}\\ \begin{array}{cc} 0, & \text{otherwise} \end{array} \end{array}.$$

The particular cases of *L*_0_, *L*_0.5_, *L*_1_ penalization are given below. Details are presented in the Appendix.

*L*_0_ **-penalization**. In the case of *L*_0_-penalization, the penalization term reflects the number of non-zero coefficients. Considering one of the optimization iterations of ALS, e.g. (12), the solution is an element-wise hard thresholding of the least square solution ${\left({\text{w}}_{j}^{1}\right)}_{LS}\;,j=1,\ldots ,\;I_{1}$ (see Appendix):


$${\left({\text{w}}_{j}^{1}\right)}_{L_{0}}=\left\{ \begin{array}{l} 0\text{ if }j\in L_{1}\text{ and ​​ ​​ }{\left({\text{w}}_{j}^{1}\right)}_{LS}\;\leq Threshold_{L_{0}}\\ \;{\left({\text{w}}_{j}^{1}\right)}_{LS}\text{ otherwise} \end{array}\right.,$$

where


$$Threshold_{L_{0}}=\frac{\sqrt{\lambda_{1}}}{|w^{3}⊗w^{2}}|.$$

*L*_0.5_**-penalization**. In the case of *L*_0.5_ penalization and considering one of the optimization steps of ALS, e.g., (12), the cost function *CostF*_*L*_0.5__ to minimize takes the following form:


(16) $$CostF_{L_{0.5}}\left({\text{w}}_{j}^{1}\right)={\left\|v_{1}^{j}-{\text{w}}_{j}^{1}{\left(w^{3}⊗w^{2}\right)}^{\text{T}}\right\|}^{2}+\lambda_{1}\sqrt{\left|{\text{w}}_{j}^{1}\right|}$$

or, equivalently,


(17) $$CostF_{L_{0.5}}\left({\text{w}}_{j}^{1}\right)={\left\|w^{3}⊗w^{2}\right\|}^{2}{\left({\left({\text{w}}_{j}^{1}\right)}_{LS}-{\text{w}}_{j}^{1}\right)}^{2}+\lambda_{1}\sqrt{\left|{\text{w}}_{j}^{1}\right|},$$

with solution,


$${\left(w_{j}^{1}\right)}_{L_{0.5}}=\left\{ \begin{array}{l} \;0,\;if\;j\in L_{1}\;and\;{\left(w_{j}^{1}\right)}_{LS}\;\leq Threshold_{L_{0.5}}\\ \text{​}argmin\;\left(CostF_{L_{0.5}}\left(0\right),\;CostF_{L_{0.5}}\left(ℬ\;\cdot \;{\left(w_{j}^{1}\right)}_{LS}\right)\;\right),\\ \;if\;j\in L_{1}\;and\;{\left(w_{j}^{1}\right)}_{LS}>Threshold_{L_{0.5}}\\ \;{\left(w_{j}^{1}\right)}_{LS}\;,\;otherwise, \end{array}\right.$$

where


$$Threshold_{L_{0.5}}=\frac{3}{4}{\left(\frac{{\text{λ}}_{1}}{\|w^{3}⊗w^{2}{\|}^{2}}\right)}^{{}^{2}\text{​​}╱\text{​}{\text{​}}_{3}\;},$$

and B is the biggest root of the cubic equation,


$$x{\left(1-x\right)}^{2}=C,$$

$$C=\frac{{\text{λ}}_{1}^{2}}{\|16w^{3}⊗w^{2}{\|}^{4}{\left({\left(w_{j}^{1}\right)}_{LS}\right)}^{3}},$$

in the interval [0; 1] (see Appendix).

*L*_1_ **-penalization**. Finally, in the case of *L*_1_ penalization, considering one optimization step of ALS optimization, e.g., (12), the solution is an element-wise soft-thresholding of the least square solution ${\left({\text{w}}_{j}^{1}\right)}_{LS}j=1,\ldots ,\;I_{1}$ (see Appendix):


$${\left(w_{j}^{1}\right)}_{L_{1}}=\left\{ \begin{array}{l} \;0\;,\;if\;j\in L_{1}\;and\;{\left(w_{j}^{1}\right)}_{LS}\;\leq Threshold_{L_{1}}\\ sign\left({\left(w_{j}^{1}\right)}_{LS}\right)\left(\left|{\left(w_{j}^{1}\right)}_{LS}\right|-Threshold_{L_{1}}\right)\;,\\ \;if\;j\in L_{1}\;and\;{\left(w_{j}^{1}\right)}_{LS}>Threshold_{L_{1}}\\ \;{\left(w_{j}^{1}\right)}_{LS}\;otherwise, \end{array}\right.$$

where


$$ThresholdL_{1}=\frac{\lambda_{1}}{{\left\|w^{3}⊗w^{2}\right\|}^{2}}.$$

#### 2.4.2. Integration to PREW-NPLS

**Figure 1:**
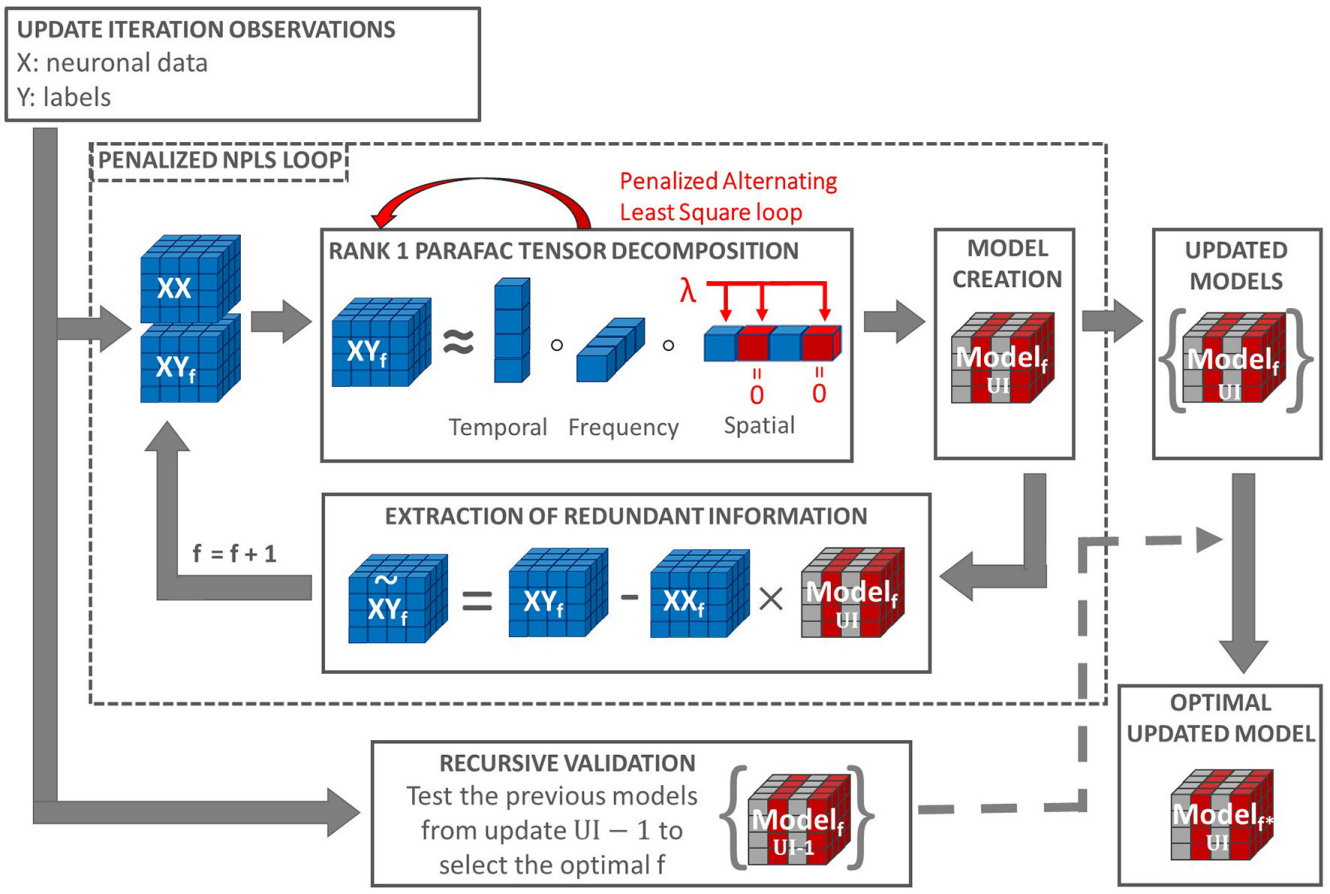
Penalized REW-NPLS (PREW-NPLS) algorithm. For the model update iteration *UI*, an updated training data set is collected. The update loop includes the recursive validation procedure for the evaluation of optimal latent variable space dimension *f* (number of factors) and penalized NPLS loop, which includes the sequential tensors **XY** and **XX** decomposition. Each step of decomposition uses Rank 1 PARAFAC, which employs penalized alternating least square procedure. Selective sparsity-promoting penalization with penalization parameter λ is applied, e.g., to the spatial dimension. Part of the weights is set to zero due to penalization (shown in red). This results in zero slices in the decoding model (shown in red). A set of updated models for all parameters *f* are stored up to the maximum *f* value, 100 in the current setting.

A Penalized PARAFAC (*R* = 1) is used in the REW-NPLS algorithm to extract a set of projectors ${\left\{w_{f}^{1}\in {\mathbb{R}}^{I_{1}},w_{f}^{2}\in {\mathbb{R}}^{I_{2}},\;\ldots ,w_{f}^{m}\in {\mathbb{R}}^{I_{m}}\right\}}_{f=1}^{F}$. For *f* = 1, all projector elements can be potentially penalized: ${ℒ}_{j,1}=\left\{1,2,\ldots ,I_{j}\right\}$, *j* = 1, …*m*. After the first set of projectors' extraction, non-zero elements of the projectors correspond to tensor slices already included in the decoding model with non-zero coefficients. For the next iterations, corresponding indexes were removed from a set of indexes to be penalized, resulting in a sequence ${ℒ}_{j,2}\subset {ℒ}_{j,1}$. A scheme representing the PREW-NPLS algorithm in the case of penalizing one data tensor direction is shown in Figure 1.

### 2.5. Experiments

This study relies on the neural signal dataset recorded during the online closed-loop BCI clinical experiments. The ≪ BCI and Tetraplegia ≫ clinical trial (NCT02550522, ClinicalTrials.gov) (University Hospital, Grenoble, 2015) was approved by French authorities: National Agency for the Safety of Medicines and Health Products (Agence nationale de sécurité du médicament et des produits de santé, ANSM) with the registration number 2015-A00650-49 and the Ethic Committee for the Protection of Individuals (Comité de Protection des Personnes, CPP) with the registration number 15-CHUG-19. The research activities were carried out in accordance with the guidelines and regulations of the ANSM and the CPP. The patient provided informed consent for the clinical trial and publication as well as to publish the information/image(s) in an online open-access publication. Details of the clinical trial protocol are available in Benabid et al. (2019).

**Figure 2:**
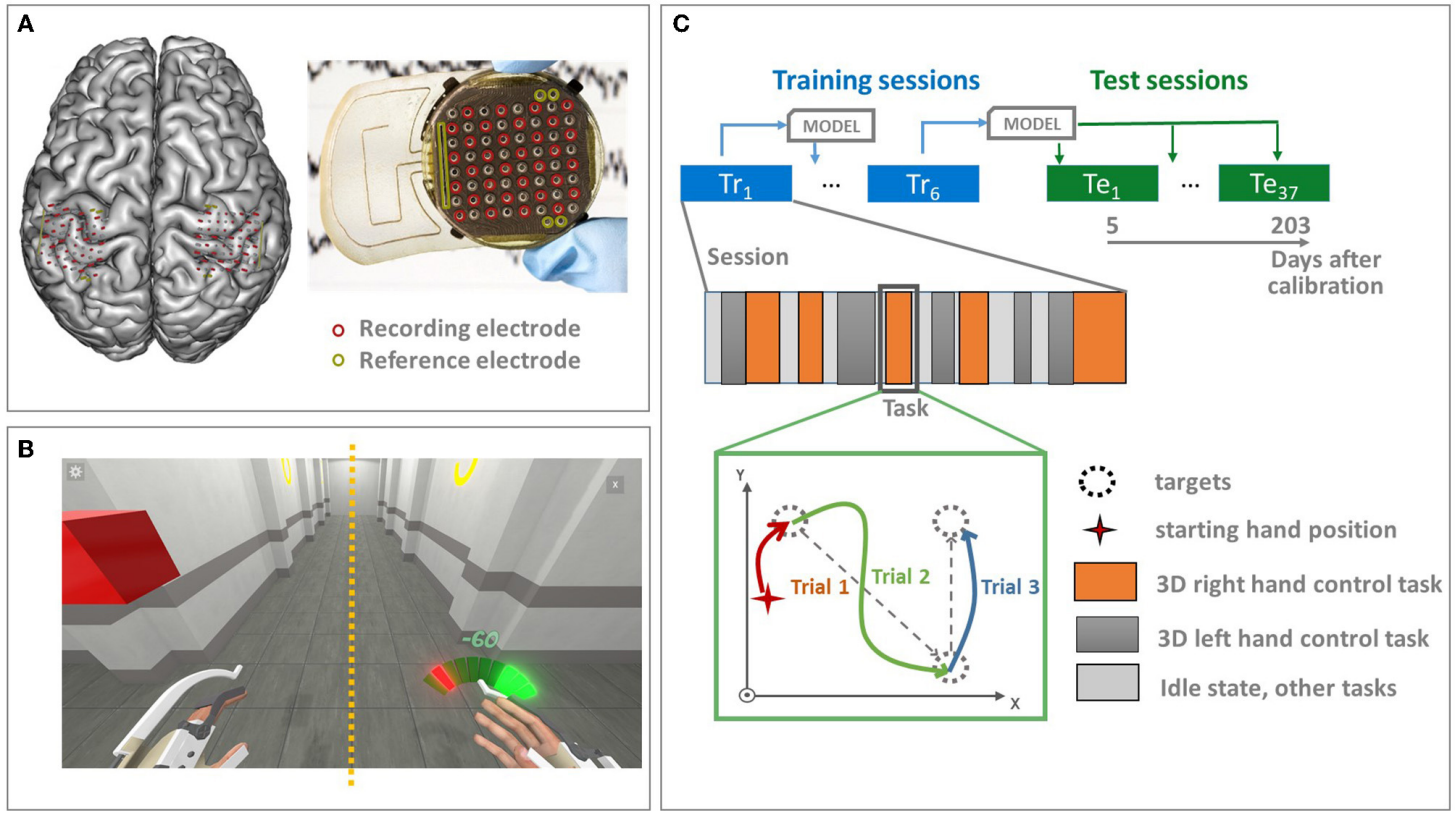
Experimental design. **(A)** The patient underwent bilateral implantation of two chronic wireless WIMAGINE implants. The recording systems were epidurally implanted within a 25 mm radial craniotomy in front of the sensory-motor cortex. The WIMAGINE implant is composed of 64 plane electrodes with a 2.3 mm diameter and a 4–4.5 mm inter-electrode distance. The digitized ECoG data from 32 electrodes from each implant (shown in red) are radio transmitted to a base station at the 586 Hz sampling rate. Reference electrodes are shown in green. **(B)** The database was recorded during the online closed-loop BCI experiments of alternative upper limbs BCI control. The virtual avatar was used as visual feedback. **(C)** The data sets were recorded in the period of 468 to 666 days after implantation and includes 43 sessions in general. The first six experimental sessions were used for incremental real-time decoder training. Separate decoders were identified to control different tasks: alternative 3D reaching tasks, and wrists rotation. They were mixed together using the Markov Mixture of expert approach (Moly et al., 2022). The decoders were tested without re-calibration with 37 experimental sessions distributed over 5–203 days after the last model update. Only 3D reaching tasks of both hands are included in the current study. The 3D reaching tasks are composed of a series of trials in which the sequentially proposed targets must be reached (pursuit tasks). The cursor position is not reset during the task, between tasks, and during the idle state.

The participant was a 29-year-old right-handed male with traumatic sensorimotor tetraplegia caused by a complete C4–C5 spinal cord injury two years prior to the study. He underwent bilateral implantation of two chronic wireless WIMAGINE implants (Benabid et al., 2019) for ECoG signal recording on 21 June 2017. Two WIMAGINE recording systems were surgically implanted into the skull near the sensory-motor cortex (SMC) through a 25-mm radial craniotomy. Before the surgery, the patients' SMC was clearly localized using functional imaging. Details are provided in Benabid et al. (2019). The WIMAGINE device is made up of an active implantable medical device composed of 64-plane platinum-iridium 90/10 electrodes with a 2.3 mm diameter and a 4–4.5 mm inter-electrode distance (Sauter-Starace et al., 2019). The recorded signals were low- and high-pass filtered, with a bandwidth range of 0.5Hz to 300Hz, using analog low-pass filters as well as a digital low-pass FIR filter embedded into the implant hardware. The digitized ECoG data from 32 electrodes from each implant (Figure 2) were radio transmitted to a custom-designed base station at a 586 Hz sampling rate.

Since the implantation, the patient had been trained to control multiple real and virtual effectors, such as games that were specially created for BCI training, a wheelchair, an exoskeleton, an avatar, and so on, using a custom-made BCI platform (Benabid et al., 2019). The database used in the study was recorded during the online closed-loop BCI experiments of upper limbs BCI control. The BCI sessions included alternative active states (AS) of the 3D reaching task for each hand, 1D wrist rotation of each hand, and the idle state (IS) (Benabid et al., 2019). The patient aimed to reach the proposed targets or rotate the wrist to specific angles following pursuit tasks. A pursuit task session was composed of successive tasks, e.g., a left-hand 3D reaching task, a right-hand 3D reaching task, IS, and so on. Each task is composed of several trials in which the cursor must reach the proposed targets. The cursor position is not reset between tasks, during the task, or during the idle state. Twenty-two targets were symmetrically distributed in two cubes in front of the patient. The virtual avatar was used as visual feedback (Benabid et al., 2019). In this study, only the left- and right-hand 3D translation trials were used for algorithm evaluation. The experimental paradigm is illustrated in Figure 2.

Data were recorded in the period of 468–666 days after implantation and included 43 sessions. The average session duration was 29 ± 8 min. During the experiments, the first six experimental sessions were used for incremental real-time decoder updates. REW NPLS decoders for each active state task were integrated into the BCI clinical trial platform. The decoders were initialized with a zero. The total training time of the decoding models was 3 h and 37 min, including a total of 189 and 194 trials of the left- and right-hand translation, respectively. The decoders were calibrated during experimental sessions in late September 2018 and were tested without re-calibration in experiments from early October 2018 to mid-March 2019, with 37 experimental sessions distributed over 5–203 days after the last model update session (468–666 days after implantation; Figure 2).

### 2.6. Application, feature extraction

During the experimental sessions, neural signal epochs of 1s for 64 recording channels $X_{t}\in {\mathbb{R}}^{586\text{x}64}$ with sliding steps of 100 ms used for feature extraction. The epochs were mapped into the feature space using a complex continuous wavelet transform (CCWT) (Morlet) with a frequency range of 10–150 Hz and a 10-Hz step. CCWT is a common feature extraction strategy that is widely used in the field of BCIs (Chao et al., 2010; Shimoda et al., 2012; Schaeffer and Aksenova, 2016; Eliseyev et al., 2017; Choi et al., 2018). The absolute value of CCWT was decimated by averaging along the temporal modality to obtain a 10-point description of 1s time epoch for each frequency band and for each channel, resulting in the temporal-frequency-spatial neural feature tensor ${\underline{X}}_{t}\in {\mathbb{R}}^{10\text{x}15\text{x}64}$. The observations of neural features *X*_*t*_ and the movement features **y**_*t*_ recorded during the decoder update/calibration sessions were used for the decoding model identification. The movement features (optimal prediction) at time *t* is defined as the 3D Cartesian vector between the current effector position and the target position (Benabid et al., 2019). The control command ***ŷ***_*t*_ for 3D hand translation is defined as the cartesian increments of the current position and is sent to the effector at 10 Hz.

### 2.7. Comparison study

To compare the proposed PREW-NPLS and the generic, non-penalized REW-NPLS algorithms, the decoding of the 3D reaching task for each hand is considered. The comparison study was restricted to penalization according to spatial modality. Three types of penalization *L*_*p*_, *p* = 0, 0.5, 1 were tested. For *L*_*p*_, *p* = 0.5, 1 penalties, a set of models were evaluated with the penalization parameter λ going from 0 to 0.5 with 0.02 steps. In the case of the *L*_0_-PREW-NPLS, preliminary results highlighted that the studied λ range was not relevant. Therefore, the models with the penalization parameter λ going from 0 to 0.05 with 0.002 steps were estimated. The cases with λ= 0 correspond to the generic, non-penalized REW-NPLS algorithm.

The performance of algorithms was evaluated offline in a pseudo-online manner. Pseudo-online simulation uses the procedure and parameters of online stream data processing. The dataset was recorded during the online closed-loop BCI experiments using the REW-NPLS decoder previously integrated into the BCI system. As the recorded data integrate the neuronal feedback into real-time decoding, the presented offline comparison study is not fully generalizable to the online case. Nevertheless, it allows the characterization of, to some extent, the studied algorithms.

To be as close as possible to the settings of the online experiments, in the comparison study, the penalized models were calibrated on the same experiments that were used for decoder training during the online closed-loop experiments.

The predicted trajectories performed during the online closed-loop experiments are related to the decoding model used during the experiments. Consequently, sample-based indicators were used to compare the predictions of the tested algorithms in the offline study. The cosine similarity indicator was based on the comparison between the predicted directions ***ŷ***_*t*_ and the optimal direction **y**_*t*_ was employed:


$$CosSim_{t}=\frac{y_{t}\cdot {\hat{y}}_{t}}{\left\|y_{t}\right\|\left\|{\hat{y}}_{t}\right\|}.$$

Here, “·” defined the dot product ***y***_*t*_ and ***ŷ***_*t*_, and they are the optimal and predicted output. The optimal 0 output is defined as the 3D Cartesian vector between the current position and the target. Notably, *CosSim*_*t*_[−1, 1] represent how direct the movement is to the final destination. A mean cosine similarity of 1 corresponds to a direct and short trajectory. *CosSim* performance criterion is evaluated as the median, 25th (Q_1_) and 75th (Q_3_) percentiles of the *CosSim*_*t*_ over samples and is notated as *CosSim* = median (Q_1_−Q_3_).

The PREW-NPLS algorithms converge to sparse solutions, fixing non-relevant model coefficients to exactly zero. The decoding performance, therefore, is not the only relevant indicator. Considering a penalized model with the penalization restricted to the *i*th dimension, the model *Sparsity* index for *i*^*th*^ dimension is introduced as the percentage of slices according to *i*th dimension of the tensor of model coefficients fixed fully to zero. For example, if the model is penalized according to the spatial dimension, minimizing the number of electrodes involved, *Sparsity* index for the spatial dimension corresponds to the percentage of electrodes fully excluded from the decoder (zero model coefficient for these electrodes for all frequencies and all time delays).

The significance of the differences in the cosine similarity between the REW-NPLS and PREW-NPLS algorithms was computed for the left and right-hand 3D translation studies and for each penalization parameter λ. The statistical analysis was performed with the non-parametric paired Wilcoxon signed rank test with (α_*multi*−*class*_ = 0.00161) and without (α = 0.05) the Bonferroni correction.

## 3. Results

**Figure 3:**
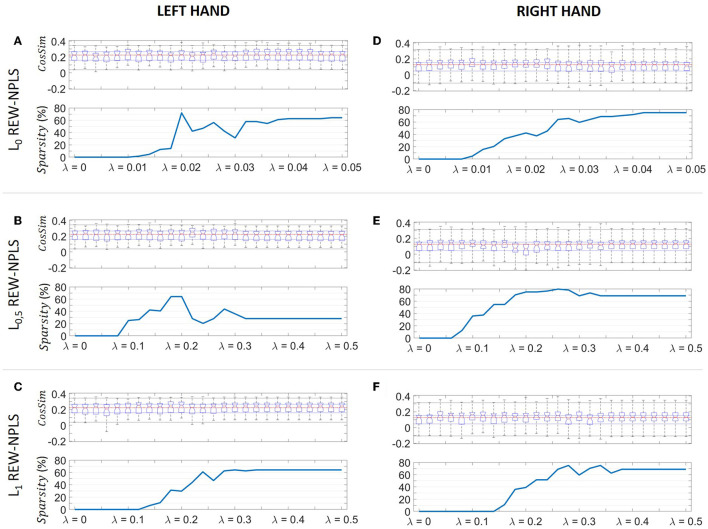
The cosine similarity and the model sparsity for the *L*_*p* penalized REW-NPLS algorithm for 3D reaching tasks decoding. The cosine similarity and the model sparsity for the *L*_0_-PREW-NPLS **(A)**, *L*_0.5_-PREW-NPLS **(B)**, and *L*_1_-PREW-NPLS **(C)** estimated for the left-hand 3D reaching task control. The cosine similarity and the model sparsity for the *L*_0_-PREW-NPLS **(D)**, *L*_0.5_-PREW-NPLS **(E)**, and *L*_1_-PREW-NPLS **(F)** estimated for the right hand 3D reaching task control. The cosine similarly is summarized using a box plot where the red line is the median the blue lines indicate the 25th and 75th percentiles (*Q*_1_ and *Q*_3_). Additionally, the whiskers show the upper and lower extreme cosine similarity obtained for the data set. The generic REW-NPLS algorithm results are presented in the first box plot of each sub-plot corresponding to λ = 0.

The cosine similarity performance and the *Sparsity* of models with *L*_*p*_, *p* = 0, 0.5, 1 penalization according to spatial modality for the left and right-hand 3D movement tasks are presented depending on the penalization coefficient λ in Figure 3. The generic REW-NPLS (λ = 0) performance is presented in the first position of each sub-figure.

The generic REW-NPLS (λ = 0) performance highlighted a *CosSim* = 0.223 (0.158 − 0.266) (median = 0.223, Q_1_ = 0.158, Q_3_ = 0.266), for the left hand, and *CosSim* = 0.127 (0.047 − 0.155) (median = 0.127, Q_1_ = 0.047, Q_3_ = 0.155) for the right-hand 3D translation decoding.

The penalized decoders showed relevant performance for different penalization parameters λ. *Sparsity* indexes generally increase with higher penalization coefficients. Decoding performance for all penalty types and penalization parameters were highly variable between sessions (high inter-session variability). Decoding of the right-hand translation stresses worse cosine similarity than decoding of the left-hand, but PREW-NPLS allowed for better decoding performance than generic REW-NPLS.

***L_0*** **PREW-NPLS** algorithm demonstrated improved performance compared to the generic REW-NPLS for different parameters λ in both hand translation and decoding tasks (Figures 3A, D). For a minor penalization, λ = 0.01, low sparsity was observed: *Sparsity* = 0% for the left hand, and *Sparsity* = 4.68% corresponding to only three electrodes removed for the right hand. Additionally, the performance reached *CosSim* = 0.252 (0.165 − 0.296) for the left hand and *CosSim* = 0.157 (0.1018 − 0.203) for the right hand translation, resulting in a median movement of the cosine similarity of 13 and 24%, respectively.

Performance improvement was demonstrated for sparser solutions using different penalization parameters. For a moderate penalty, λ = 0.026, cosine similarity *CosSim* = 0.248 (0.173 − 0.288) was achieved with a *Sparsity* = 56.25% for the left hand. For the right hand, with λ = 0.018, cosine similarity *CosSim* = 0.157 (0.0989 − 0.185) was achieved with *Sparsity* = 37.5%. A sparser solution with *Sparsity* = 45.31% was obtained for the right hand for λ = 0.024, with *CosSim* = 0.153 (0.0786 − 0.198).

For a strong penalty (0.04 ≤ λ ≤ 0.046), *L_0* PREW-NPLS demonstrated for the left hand a similar decoding performance *CosSim* = 0.248 (0.162 − 0.294) with 40 electrode parameters set to the 0 value, *Sparsity* = 62.5%. For the right hand, *L_0* PREW-NPLS models converged to a highly sparse solution with λ> 0.046 with 48 electrodes over 64 removed (*Sparsity* = 75%), which highlighted decoding performance similar to the generic REW-NPLS [*CosSim* = 0.128 (0.058 − 0.168)].

Similarly, the ***L_0.5*** **PREW-NPLS** decoder demonstrated better decoding performance with sparser solutions for several penalization parameters compared to the generic REW-NPLS decoder (Figures 3B, E).

In particular, for a moderate penalty, λ = 0.22, for the left hand, 18 electrode parameter weights were set to zero (*Sparsity* = 28.13%), achieving higher performance *CosSim* = 0.253 (0.189 − 0.301) compared to the generic REW-NPLS model. Similarly, for the right hand, for penalization λ = 0.16, a sparse model (*Sparsity* = 54.69%) was identified, increasing the decoding performance *CosSim* = 0.150 (0.0881 − 0.176). Sparsest models were obtained for λ = 0.26, 0.28 with *Sparsity* = 79.69% and *Sparsity* = 78.13%, respectively.

With higher penalization, for the left hand, the performance *CosSim* = 0.245 (0.156 − 0.2838) was achieved with *Sparsity* = 35.94% for λ = 0.3. For λ ≥ 0.32, the sparsity converged to *Sparsity* = 28.13% with decoding performance similar to the generic REW-NPLS model: *CosSim* = 0.217 (0.143 − 0.261). For the right hand, for λ ≥ 0.36, the models converged to the same solutions with *Sparsity* = 68.75% (44 electrodes removed) and a cosine similarity *CosSim* = 0.131 (0.0835 − 0.186) slightly superior compared to the generic REW-NPLS decoder.

***L_1*** **PREW-NPLS** decoder demonstrated similar results (Figures 3C, F). For low penalty, λ = 0.12, *Sparsity* = 0%, the *L_1* PREW-NPLS model highlighted *CosSim* = 0.253 (0.151 − 0.286) for the left hand. For the right hand, the models with small penalization parameters λ = 0.04, 0.06 and 0.1 with zero sparsity (*Sparsity* = 0 %) highlighted a cosine similarity *CosSim* = 0.154 (0.0915 − 0.202), *CosSim* = 0.158 (0.0791 − 0.184) and *CosSim* = 0.164 (0.0959 − 0.191) representing an improvements of 21, 24, and 29%, respectively.

For higher penalization parameters, *Sparsity* = 29.69% was reached for λ = 0.20 with a decoding performance of *CosSim* = 0.249(0.162 − 0.295) for the left hand. Similarly, for the right hand, the decoding performance *CosSim* = 0.154 (0.101 − 0.192) and *CosSim* = 0.152 (0.0872 − 0.197) with 33 (51.56%) and 44 (68.75%) electrode parameters weights set to zero was achieved for λ = 0.22, 0.26, respectively.

Finally, for the strong penalization, λ ≥ 0.34, 41 electrode parameter weights were set to zero leading to *CosSim* = 0.245 (0.173 − 0.283) for the left-hand. For penalization parameters λ ≥ 0.38, the models stabilized to a solution with *Sparsity* = 68.75% with a decoding performance *CosSim* = 0.131 (0.0835 − 0.186) for the right hand.

The REW-NPLS and the *L_p* PREW-NPLS model parameter weights were shown in the temporal, frequency, and spatial domains in Supplementary Figures 1, 2 for the left- and right-hand translation models, respectively. The presented models were the ones with the maximal penalization coefficient λ=0.06,0.4 and 0.4 for the *L_0-, L_0.5,-* and *L_1* PREW-NPLS algorithms, respectively. The spatial parameter weights are presented in Supplementary Figures 3, 4 on a map with the electrode locations relative to the sensory (SS) and motor (MS) sulci.

The results of the statistical analysis performed with the non-parametric paired Wilcoxon signed rank test with and without the Bonferroni correction are presented in Supplementary Tables 1, 2 for the left- and right-hand translation and decoding for each of the PREW-NPLS algorithms. Numerous models highlighted the statistically significant performance improvement of the proposed PREW-NPLS algorithms compared to the generic REW-NPLS for all the penalization types.

## 4. Discussion

Among the various potential applications, the functional compensation/restoration of individuals suffering from severe motor disabilities has always been a focus of BCI research. The primary challenge of motor BCIs is the high-dimensional control of complex effectors. To achieve this objective, high-resolution neuronal activity recording is generally required, which results in the high-dimensional data flow being processed in real time with a high decision rate of at least (8–10 Hz). Moreover, closed-loop decoder training is reported to be more effective (Jarosiewicz et al., 2013) compared to decoders trained classically using training data recorded in an open-loop setting. CLDA BCIs use incremental learning/adaptive algorithms capable of updating a decoder in real time during BCI sessions, increasing the computational load. In addition to high computing time and power requirements, high-dimensional feature space may lead to numerous issues, complicating decoder training. Efficient feature selection decreases the feature space dimension and may allow for improving the generalization ability of the decoder by excluding irrelevant, redundant, or noisy variables. As neuronal activity features are naturally grouped (e.g., by the sensors and the frequency bands), group-wise feature selection may be beneficial, which can help fully exclude non-informative or redundant electrodes or/and frequency bands from consideration. A group-wise feature selection procedure embedded in adaptive/incremental learning of the decoder is proposed in the study. It was based on sparsity-promoting penalization of the data tensor decomposition integration to the REW-NPLS algorithm. *L*_*p*_, *p* = 0, 0.5, 1 norm/pseudo-norm penalties were studied. Sparsity-promoting penalties were applied to feature groups corresponding to slices of the data tensor related to the mode of analysis (e.g., spatial).

The proposed algorithms were tested with a dataset of left/right arms 3D translations of a virtual avatar controlled by the tetraplegic subject. The studied models were trained on the same data (the first six sessions), which were used for decoder training during the online experiments. The number of training sessions was relatively small (14%) and focused at the beginning of the experiments. This may partially explain the high inter-session variability of the decoding performance for all the algorithms (depicted in Supplementary Figure 5). However, *L*_*p*_*-*PREW-NPLS algorithms highlighted equal or better decoding performance compared to the generic REW-NPLS decoder with sparse solutions, with up to two-thirds of the electrode parameter weights set to zero for both tasks of the left and the right-hand translation. Computational load measurements demonstrated the expected decrease (close to linear) in memory consumption and computational time during the model execution stage, depending on the model sparsity for 3D hand translation. 25, 50, and 75% of sparsity in the spatial dimension results in an average of 25, 50, and 75% decreases in memory consumption and a 23, 45, and 67% decrease in computational time.

The PREW-NPLS decoders highlighted equal or better decoding performance compared to the generic REW-NPLS algorithm for the majority of the penalization parameters. For example, for the right-hand decoding, the *L*_1_-PREW-NPLS decoder with a moderate penalization parameter λ = 0.26, highlighted a significant (*p*−*value* = 10^−6^) cosine similarity improvement of 21, 116, and [[Mathtype-mtef1-eqn-317.mtf]] for the median and the 25th and 75th percentiles, respectively, with fewer than half of the electrodes, maintained at a non-zero value (33 electrodes set to zero). For the small penalization parameters (λ = 0.1), the model converged to a non-sparse solution with a significant cosine similarity improvement (*p*−*value* = 10^−6^), leading to a median, 25th and 75th percentile enhancement of 24, 104, and 23%, respectively. The sparsest solution with *the L*_1_-PREW-NPLS algorithm removed 75% of the electrodes without decreasing the cosine similarity indicator and reducing the features space from 10 × 15 × 64 = 9, 600 features to 10 × 15 × 16 = 2, 400 features.

Decoding performance improvements were more evident for the 3D right-hand translation models than the left-hand 3D translation. As the 3D right-hand translation models demonstrated lower decoding performance than left-hand translation models, a sparse decoder is more effective for a less performant neural signal decoder in this study.

An increase in the training dataset size may potentially improve decoder performance and limit overtraining. Supplementary Figure 6 shows the cosine similarity and model sparsity using *L*_1_*-*PREW-NPLS with larger training datasets: 30 and 50% of the whole data (13 and 22 sessions, respectively). It results in fewer sparse models and an improvement in the decoder's performance (Supplementary Figure 6). However, in BCI research, the recording of data is highly expensive, limiting access to big datasets. The decoder training method with shorter recordings is highly profitable for real-life BCI applications.

The relative efficiency of penalization types is unclear in the current study. The maximum improvement in performance was achieved using *L*_1_ penalization, but the results are comparable to other penalization types. Application *L*_0.5_ penalty is computationally heavier compared to *L*_0_ or *L*_1_ penalties without evident advantages. A complimentary comparative study may allow clarification of the relative efficiency of the proposed penalizations.

*L*_0_*-*PREW-NPLS and *L*_1_*-*PREW-NPLS algorithms are sufficiently computationally effective to be applied for the online closed-loop decoder adaptation. To reproduce the online experimental conditions, a pseudo-online simulation was conducted using the same parameters (buffer size, batch training, and so on) as used in real time to ensure the compatibility of the proposed algorithm with the real-time application.

Finally, reducing the feature space dimension may lead to more interpretable models. Several activation patterns were discernible. In the spatial domains, the electrodes close to the motor and sensory sulci exhibit important parameter weights.

The current study is limited to offline simulations using datasets recorded from previous online closed-loop experiments with the REW-NPLS decoder integrated into the BCI system. As the neuronal signal integrates the neuronal feedback to the decoder, which was applied in real time, the offline comparison study is not fully generalizable. However, conducting real experiments to compare all algorithms is too costly. In addition, as the neuronal feedback is related to the real-time decoder output, the comparison results are likely to be biased in favor of the decoder applied during real-time experiments rather than the newly tested algorithms.

The study presented in the study is limited to penalization in the spatial domain, which is crucial for decreasing data transfer. In order to evaluate the influence on the decoding performance of penalization applied to other modalities, further research needs to be conducted to include decoders that promote sparsity in the frequency and temporal domains.

This study reports a case study. The results are obtained from the dataset, which was based on a single-subject clinical trial. Further tests with more subjects are required to support the conclusions. The authors plan to contribute to conducting clinical trials to assess the proposed algorithm in real-time experiments.

## Data availability statement

The data analyzed in this study is subject to the following licenses/restrictions. Data used for this study are from recordings from the clinical trial (ClinicalTrials.gov, NCT02550522) and are not publicly available. Part of the dataset may be provided upon reasonable request. Requests to access these datasets should be directed at: tetiana.aksenova@cea.fr.

## Ethics statement

The studies involving human participants were reviewed and approved by National Agency for the Safety of Medicines and Health Products (Agence nationale de sécurité du médicament et des produits de santé: ANSM), registration number 2015-A00650-49, and the Committee for the Protection of Individuals (Comité de Protection des Personnes—CPP), registration number 15-CHUG-19. The patients/participants provided their written informed consent to participate in this study.

## Author contributions

AM, AA, and TA: designed the algorithm. AM and AA: helped with using software. AM and FM: analyzed the data. AM, AA, FM, and TA: wrote the manuscript. All authors contributed to the article and approved the submitted version.

## Supplementary material

Supplementary Table 1 Significance of the performance (*CosSimmedian*) differences between the REW-NPLS and the L_0, L_0.5, and L_1 PREW-NPLS algorithms for the left hand 3D translation study using the non-parametric paired Wilcoxon signed rank test with and without the Bonferroni correction (α = 0.05 and α_*Bonferroni*_ = 0.00161).

Supplementary Table 2 Significance of the performance (*CosSimmedian*) differences between the REW-NPLS and the L_0, L_0.5, and L_1 PREW-NPLS algorithms for the right hand 3D translation study using the non-parametric paired Wilcoxon signed rank test with and without the Bonferroni correction (α = 0.05 and α_*Bonferroni*_ = 0.00161).

Supplementary Figure 1 Parameter weights of the generic REW-NPLS and *L*_*p*_, *p* = 0, 0.5, 1 PREW-NPLS for the left-hand 3D translation. Absolute values of model coefficients are projected into spatial, frequency, or temporal domains. The parameter weights related to the three controlled axes (*y*_1_, *y*_2_, and *y*_3_) are represented using blue, orange, and yellow lines, respectively.

Supplementary Figure 2 Parameter weights of the generic REW-NPLS and *L*_*p*_, *p* = 0, 0.5, 1, PREW-NPLS for the right-hand 3D translation. Absolute values of model coefficients are projected into spatial, frequency, or temporal domains. The parameter weights related to the three controlled axes (*y*_1_, *y*_2_, and *y*_3_) are represented using blue, orange, and yellow lines, respectively.

Supplementary Figure 3 Parameter weights of the models identified using generic REW-NPLS and *L*_*p*_, *p* = 0, 0.5, 1, PREW-NPLS algorithms (the left-hand 3D translation study) projected into the spatial domain depending on the electrode locations in the implant. The sensory sulcus (SS) and motor sulcus (MS) are shown by yellow and red curves respectively.

Supplementary Figure 4 Parameter weights of the models identified using generic REW-NPLS and *L*_*p*_, *p* = 0, 0.5, 1, PREW-NPLS algorithms (the right-hand 3D translation study) projected into the spatial domain depending on the electrode locations in the implant. The sensory sulcus (SS) and motor sulcus (MS) are shown by yellow and red curves respectively.

Supplementary Figure 5 The cosine similarity for the *L*_*p*_ penalized REW-NPLS algorithms (*p* = 0, 0.5, 1), for different λ for 37 test sessions. Cosine similarity of decoding of left-hand 3D translation is depicted in subplots **(A–C)**. Cosine similarity of decoding of right-hand 3D translation is depicted in subplots **(D–F)**.

Supplementary Figure 6 The cosine similarity and the model sparsity for the L_1 penalized REW-NPLS algorithm for 3D reaching tasks using 30% **(A, C)** and 50% **(B, D)** of the data set for the decoder training. The cosine similarly is summarized using a box plot where the red line is the median the blue lines indicate the 25th and 75th percentiles (*Q*_1_ and *Q*_3_). Additionally, the whiskers show the upper and lower extreme cosine similarity obtained for the data set. The generic REW-NPLS algorithm results are presented in the first box plot of each sub-plot corresponding to λ = 0.
