# Supplementary material for: Online adaptive group-wise sparse Penalized Recursive Exponentially Weighted N-way Partial Least Square for epidural intracranial BCI
Source: Front Hum Neurosci. 2023 Mar 6;17:1075666. doi: 10.3389/fnhum.2023.1075666 (PMC10025377; doi:10.3389/fnhum.2023.1075666)
Supplement: Supplementary file 1 [file Data_Sheet_1.zip › Appendix.docx]

# PREW-NPLS Appendix

The detailed description of an ALS iteration for $L_{p}, p=1,2,3$ penalization is presented in the Appendix.

**Penalization** $\boldsymbol{L}_{\boldsymbol{0}}$**.** Let us consider one of optimization step, e.g. (12), of ALS in case of $L_{0}$ penalization. The cost function ${CostF}_{L_{0}}$ to minimize takes the form:

| ${CostF}_{L_{0}}\left( w_{j}^{1} \right)=\left\Vert\mathbf{v}_{1}^{j}\boldsymbol{-}w_{j}^{1}\left( \mathbf{w}^{3}\otimes\mathbf{w}^{2} \right)^{T} \right\Vert^{2}+\lambda_{1}\left( {1-\delta}_{0,w_{j}^{1}} \right) \to min.$ | (A1) |
| --- | --- |

Here $\delta_{0,w_{j}^{1}}=1$ if $w_{j}^{1}=0$, and $\delta_{0,w_{j}^{1}}=0$ otherwise. The solution of (A1) is either $w_{j}^{1}=0$ or, if $w_{j}^{1}\neq0$, then $\delta_{0,w_{j}^{1}}=0,$ and

| ${CostF}_{L_{0}}\left( w_{j}^{1} \right)=\left\Vert\mathbf{v}_{1}^{j}\boldsymbol{-}w_{j}^{1}\left( \mathbf{w}^{3}\otimes\mathbf{w}^{2} \right)^{T} \right\Vert^{2} +\lambda_{1}\to min,$ |  |
| --- | --- |
| $\underset{w_{j}^{1}}{\mathrm{argmin}} \left( \left\Vert\mathbf{V}_{1}^{j}\boldsymbol{-}w_{j}^{1}\left( \mathbf{w}^{3}\otimes\mathbf{w}^{2} \right)^{T} \right\Vert^{2}+\lambda_{1} \right)=\underset{w_{j}^{1}}{\mathrm{argmin}} \left( \left\Vert\mathbf{v}_{1}^{j}\boldsymbol{-}w_{j}^{1}\left( \mathbf{w}^{3}\otimes\mathbf{w}^{2} \right)^{T} \right\Vert^{2} \right)$. |  |

The LS solution of non-penalized task $\left( w_{j}^{1} \right)_{LS}\boldsymbol{=}\frac{\mathbf{v}_{1}^{j}\left( \mathbf{w}^{3}\otimes\mathbf{w}^{2} \right)}{\left\| \mathbf{w}^{3}\otimes\mathbf{w}^{2} \right\|^{2}}$. In order to choose the solution of (A1) one may compare ${CostF}_{L_{0}}\left( O \right)$ and ${CostF}_{L_{0}}\left( \left( w_{j}^{1} \right)_{LS} \right)={CostF}_{L_{0}}\left( \frac{\mathbf{v}_{1}^{j}\left( \mathbf{w}^{3}\otimes\mathbf{w}^{2} \right)}{\left\| \mathbf{w}^{3}\otimes\mathbf{w}^{2} \right\|^{2}} \right)$. Here ${CostF}_{L_{0}}\left( O \right)=\left( \mathbf{v}_{1}^{j} \right)^{2}$. The second candidate ${CostF}_{L_{0}}\left( \left( w_{j}^{1} \right)_{LS} \right)$ takes the form

| ${CostF}_{L_{0}}\left( \left( w_{j}^{1} \right)_{LS} \right)={CostF}_{L_{0}}\left( O \right)-\left\Vert\mathbf{w}^{3}\otimes\mathbf{w}^{2} \right\Vert^{2}\left( \left( w_{j}^{1} \right)_{LS} \right)^{2}\boldsymbol{+} \lambda_{1}$ | (A2) |
| --- | --- |

From (A2), it follows that $L_{0}$ penalization correspond to hard thresholding of the least square solution $\left( w_{j}^{1} \right)_{LS}$:

| $\left( w_{j}^{1} \right)_{L_{0}}=argmin {CostF}_{L_{0}}\left( w_{j}^{1} \right)=\left\{ \begin{matrix} 0, \mathrm{if} i\in\mathcal{L}_{1} \mathrm{and} \left( w_{j}^{1} \right)_{LS} \leq ThresholdL_{0} \\ \left( w_{j}^{1} \right)_{LS} \mathrm{otherwise} \end{matrix} \right.$ , |  |
| --- | --- |
| $ThresholdL_{0}=\frac{\sqrt{\lambda_{1}}}{\left\Vert\mathbf{w}^{3}\otimes\mathbf{w}^{2} \right\Vert}$ . |  |

Other optimization steps allow similar consideration.

**Penalization** $\boldsymbol{L}_{\boldsymbol{1}}$**.** An optimization step, e.g. (12), of ALS optimization in case of $L_{1}$ penalization takes the form

| $\left\Vert\mathbf{v}_{1}^{j}\boldsymbol{-}w_{j}^{1}\left( \mathbf{w}^{3}\otimes\mathbf{w}^{2} \right)^{T} \right\Vert^{2}+\lambda_{1}\left\vert w_{j}^{1} \right\vert\to min.$ | (A3) |
| --- | --- |

In an inner-product space, the generalized Pythagorean theorem states that squared norm of sum of two orthogonal vectors is equal to sum of their squared norms [1]:

| $\left\Vert\mathbf{v}_{1}^{j}\boldsymbol{-}w_{j}^{1}\left( \mathbf{w}^{3}\otimes\mathbf{w}^{2} \right)^{T} \right\Vert^{2}=\left\Vert\mathbf{v}_{1}^{j}\boldsymbol{-}\left( w_{j}^{1} \right)_{LS}\left( \mathbf{w}^{3}\otimes\mathbf{w}^{2} \right)^{T} \right\Vert^{2}+\left\Vert\left( w_{j}^{1} \right)_{LS}\left( \mathbf{w}^{3}\otimes\mathbf{w}^{2} \right)^{T}-w_{j}^{1}\left( \mathbf{w}^{3}\otimes\mathbf{w}^{2} \right)^{T} \right\Vert^{2},$ |  |
| --- | --- |

due to orthogonality (Figure 4). As only second term depend on the variable to optimize, the cost function takes the form:

| ${{CostF}_{L_{1}}\left( w_{j}^{1} \right)=\left\Vert\left( w_{j}^{1} \right)_{LS}\left( \mathbf{w}^{3}\otimes\mathbf{w}^{2} \right)^{T}-w_{j}^{1}\left( \mathbf{w}^{3}\otimes\mathbf{w}^{2} \right)^{T} \right\Vert}^{2}+\lambda_{1}\left\vert w_{j}^{1} \right\vert\to min.$ | (A4) |
| --- | --- |

Let us consider the case when scalar product of $\mathbf{v}_{1}^{j}$and $\left( \mathbf{w}^{3}\otimes\mathbf{w}^{2} \right)^{T}$ is non-negative, and $w_{j}^{1}\geq0$, and the opposite case when $w_{j}^{1}\leq0$. In the first case

| $\left\Vert\left( w_{j}^{1} \right)_{LS}\left( \mathbf{w}^{3}\otimes\mathbf{w}^{2} \right)^{T}-w_{j}^{1}\left( \mathbf{w}^{3}\otimes\mathbf{w}^{2} \right)^{T} \right\Vert^{2}+\lambda_{1}w_{j}^{1} \to min.$ | (A5) |
| --- | --- |

As vectors $\left( w_{j}^{1} \right)_{LS}\left( \mathbf{w}^{3}\otimes\mathbf{w}^{2} \right)^{T}$ and $w_{j}^{1}\left( \mathbf{w}^{3}\otimes\mathbf{w}^{2} \right)^{T}$ are collinear and have the same direction, triangle inequality for norm degrade to equality

| $\left( \left( w_{j}^{1} \right)_{LS}\left\Vert\mathbf{w}^{3}\otimes\mathbf{w}^{2} \right\Vert-w_{j}^{1}\left\Vert\mathbf{w}^{3}\otimes\mathbf{w}^{2} \right\Vert\right)^{2}+\lambda_{1}w_{j}^{1} \to min.$ | (A6) |
| --- | --- |

After differentiation and taking into account that $w_{j}^{1}\geq0$,

| $w_{j}^{1}=\left\{ \begin{matrix} 0,\mathrm{if} \lambda_{1}\geq\left( w_{j}^{1} \right)_{LS}\left\Vert\mathbf{w}^{3}\otimes\mathbf{w}^{2} \right\Vert^{2} \\ \frac{\left( w_{j}^{1} \right)_{LS}\left\Vert\mathbf{w}^{3}\otimes\mathbf{w}^{2} \right\Vert^{2}-\lambda_{1}}{\left\Vert\mathbf{w}^{3}\otimes\mathbf{w}^{2} \right\Vert^{2}} \mathrm{otherwise} \end{matrix} \right.$. |  |
| --- | --- |

Second case $w_{j}^{1}<0$is considered similarly and resulting in soft thresholding:

| $\left( w_{j}^{1} \right)_{L_{1}}=argmin {CostF}_{L_{1}}\left( w_{j}^{1} \right)=\left\{ \begin{matrix} 0, \mathrm{if} j\in\mathcal{L}_{1} \mathrm{and} \left( w_{j}^{1} \right)_{LS} \leq ThresholdL_{1} \\ sign\left( \left( w_{j}^{1} \right)_{LS} \right)\left( \left\vert\left( w_{j}^{1} \right)_{LS} \right\vert-ThresholdL_{1} \right)\mathrm{if} i\in\mathcal{L}_{1} \mathrm{and} \left( w_{j}^{1} \right)_{LS}>ThresholdL_{1} \\ \left( w_{j}^{1} \right)_{LS} \mathrm{otherwise} \end{matrix} \right.$ , |
| --- |
| $ThresholdL_{1}=\frac{\lambda_{1}}{\left\Vert\mathbf{w}^{3}\otimes\mathbf{w}^{2} \right\Vert^{2}}$ . |

**Penalization** $\boldsymbol{L}_{\boldsymbol{0.5}}$**.** An optimization step, e.g. (12), of ALS optimization in case of $L_{0.5}$ penalization takes the form

| $\left\Vert\mathbf{v}_{1}^{j}\boldsymbol{-}w_{j}^{1}\left( \mathbf{w}^{3}\otimes\mathbf{w}^{2} \right)^{T} \right\Vert^{2}+\lambda_{1}\sqrt{\left\vert w_{j}^{1} \right\vert} \to min .$ | (A7) |
| --- | --- |

Similarly to (A4),

| ${{CostF}_{L_{0.5}}\left( w_{j}^{1} \right)=\left( \left( w_{j}^{1} \right)_{LS}\left\Vert\mathbf{w}^{3}\otimes\mathbf{w}^{2} \right\Vert-w_{j}^{1}\left\Vert\mathbf{w}^{3}\otimes\mathbf{w}^{2} \right\Vert\right)}^{2}+\lambda_{1}\sqrt{\left\vert w_{j}^{1} \right\vert}=$  ${=\left\Vert\mathbf{w}^{3}\otimes\mathbf{w}^{2} \right\Vert^{2}\left( \left( w_{j}^{1} \right)_{LS}-w_{j}^{1} \right)}^{2}+\lambda_{1}\sqrt{\left\vert w_{j}^{1} \right\vert}$ | (A8) |
| --- | --- |

Let us consider the case if scalar product of $\mathbf{v}_{1}^{j}$and $\left( \mathbf{w}^{3}\otimes\mathbf{w}^{2} \right)^{T}$ is non-negative, and $w_{j}^{1}\geq0$. Taking into account that $w_{j}^{1}\leq\left( w_{j}^{1} \right)_{LS}$ , the interval of interest is $\left[ 0,\left( w_{j}^{1} \right)_{LS} \right]$. For $w_{j}^{1}>0$ the derivatives of cost function are

| $\left( {CostF}_{L_{0.5}}\left( w_{j}^{1} \right) \right)^{'}={2\left\Vert\mathbf{w}^{3}\otimes\mathbf{w}^{2} \right\Vert}^{2}\left( \left( w_{j}^{1} \right)_{LS}-w_{j}^{1} \right)+\frac{\lambda_{1}}{2\sqrt{w_{j}^{1}}}$ ,  $\left( {CostF}_{L_{0.5}}\left( w_{j}^{1} \right) \right)^{''}={2\left\Vert\mathbf{w}^{3}\otimes\mathbf{w}^{2} \right\Vert}^{2}-\frac{\lambda_{1}}{4\left( w_{j}^{1} \right)^{\frac{3}{2}}} .$ | (A9) |
| --- | --- |

Let us note that if $\left( {CostF}_{L_{0.5}}\left( w_{j}^{1} \right) \right)^{'}>0$ for $w_{j}^{1}\in\left[ 0,\left( w_{j}^{1} \right)_{LS} \right]$, ${CostF}_{L_{0.5}}\left( w_{j}^{1} \right)$ is monotonically increasing function at the interval $\left[ 0,\left( w_{j}^{1} \right)_{LS} \right]$ and has minimum at $w_{j}^{1}=0$. First, let us study the cases when first derivative of the cost function is positive. Let us note that $\left( {CostL}_{0.5}\left( w_{j}^{1} \right) \right)^{''}$is monotonically increase. $\left( {CostF}_{L_{0.5}}\left( w_{j}^{1} \right) \right)^{''}<0$ for $w_{j}^{1}\in\left[ 0,\left( w_{j}^{1} \right)_{LS} \right]$ if ${\lambda_{1}>8\left\| \mathbf{w}^{3}\otimes\mathbf{w}^{2} \right\|}^{2}\left( \left( w_{j}^{1} \right)_{LS} \right)^{\frac{3}{2}}$. For negative second derivative first derivative is monotonically decrease. As at the end point $\left( {CostF}_{L_{0.5}}\left( \left( w_{j}^{1} \right)_{LS} \right) \right)^{'}>0$, derivative is positive for all interval of interest and optimal $\left( w_{j}^{1} \right)_{L_{0.5}}=0$ (Figure 5 (A)). Otherwise $\left( {CostF}_{L_{0.5}}\left( w_{j}^{1} \right) \right)^{''}=0$ at $\hat{w}_{j}^{1}=\frac{\left( \lambda_{1} \right)^{\frac{2}{3}}}{{4\left\| \mathbf{w}^{3}\otimes\mathbf{w}^{2} \right\|}^{\frac{2}{3}}}$ . The derivative $\left( {CostF}_{L_{0.5}}\left( w_{j}^{1} \right) \right)^{'}$ decreases if $w_{j}^{1}\in\left[ 0,\hat{w}_{j}^{1} \right]$ and increases $w_{j}^{1}\in\left[ \hat{w}_{j}^{1},\left( w_{j}^{1} \right)_{LS} \right]$. Moreover $\left( {CostF}_{L_{0.5}}\left( \hat{w}_{j}^{1} \right) \right)^{'}>0$ if ${\lambda_{1}>\frac{8}{3\sqrt{3}}\left\| \mathbf{w}^{3}\otimes\mathbf{w}^{2} \right\|}^{2}\left( \left( w_{j}^{1} \right)_{LS} \right)^{\frac{3}{2}}$. In this case optimal solution is still $\left( w_{j}^{1} \right)_{{LS\_L}_{0.5}}=0$ (Figure 5 (B)).

If $\left( {CostF}_{L_{0.5}}\left( \hat{w}_{j}^{1} \right) \right)^{'}<0$ the cost function ${CostF}_{L_{0.5}}\left( w_{j}^{1} \right)$ has 2 local extrema located in $\left[ 0,\hat{w}_{j}^{1} \right]$ and $\left[ \hat{w}_{j}^{1},\left( w_{j}^{1} \right)_{LS} \right]$ (Figure 5 (C)). Only second one may correspond to the minimum of cost function (Figure 5 (C)). It may be computed explicitly from $\left( {CostF}_{L_{0.5}}\left( w_{j}^{1} \right) \right)^{'}=0$. Squared, it may be viewed as cubic equation for $x= \frac{w_{j}^{1}}{\left( w_{j}^{1} \right)_{LS}}$

| $x{(1-x)}^{2}=C$ , $C=\frac{\lambda_{1}^{2}}{16\left\Vert\mathbf{w}^{3}\otimes\mathbf{w}^{2} \right\Vert^{4}\left( \left( w_{j}^{1} \right)_{LS} \right)^{3}}$ . | (A10) |
| --- | --- |

The solution of (A10) in the interval of interest $w_{j}^{1}\in\left[ 0,\left( w_{j}^{1} \right)_{LS} \right]$ ($x\in\left[ 0,1 \right]$) exist in the case under considration as $C\in\left[ 0,\frac{4}{27} \right]$ if ${\lambda_{1}\leq\frac{8}{3\sqrt{3}}\left\| \mathbf{w}^{3}\otimes\mathbf{w}^{2} \right\|}^{2}\left( \left( w_{j}^{1} \right)_{LS} \right)^{\frac{3}{2}}$. By the properties of the function (Figure 5 (C)), the minimum of cost function may be a biggest root of (A10) in the interval [0; 1] denoted $x^{*}$. The minimum is attained at one of the points: 0 or $x^{*}\left( w_{j}^{1} \right)_{LS}$. Distinguishing between these options is straightforward. As a result,

| $\left( w_{j}^{1} \right)_{L_{0.5}}=argmin{CostF}_{L_{0.5}}\left( w_{j}^{1} \right)=\left\{ \begin{matrix} 0, \mathrm{if} j\in\mathcal{L}_{1} \mathrm{and} \left( w_{j}^{1} \right)_{LS} \leq ThresholdL_{0.5} \\ argmin \left( {CostF}_{L_{0.5}}\left( 0 \right),{CostF}_{L_{0.5}}\left( x^{*}\cdot\left( w_{j}^{1} \right)_{LS} \right) \right), if i\in\mathcal{L}_{1} \mathrm{and} \left( w_{j}^{1} \right)_{LS}>ThresholdL_{0.5} \\ \left( w_{j}^{1} \right)_{LS} \mathrm{otherwise} \end{matrix} \right.$ , |  |
| --- | --- |
| $ThresholdL_{0.5}=\frac{3}{4}\left( \frac{\lambda_{1}}{\left\Vert\mathbf{w}^{3}\otimes\mathbf{w}^{2} \right\Vert^{2}} \right)^{\frac{2}{3}}$ , |  |

and $x^{*}$ is a biggest root of (A10) in the interval [0; 1].

Figure 4: Optimization problem (A3) of ALS iterations in case of $\boldsymbol{L}_{\boldsymbol{1}}$ penalization is simplified due to orthogonality of terms $\left( \boldsymbol{v}_{\boldsymbol{1}}^{\boldsymbol{j}}\boldsymbol{-}\left( \boldsymbol{w}_{\boldsymbol{j}}^{\boldsymbol{1}} \right)_{\boldsymbol{LS}}\left( \boldsymbol{w}^{\boldsymbol{3}}\boldsymbol{\otimes}\boldsymbol{w}^{\boldsymbol{2}} \right)^{\boldsymbol{T}} \right)$ and $\left( \left( \boldsymbol{w}_{\boldsymbol{j}}^{\boldsymbol{1}} \right)_{\boldsymbol{LS}}\left( \boldsymbol{w}^{\boldsymbol{3}}\boldsymbol{\otimes}\boldsymbol{w}^{\boldsymbol{2}} \right)^{\boldsymbol{T}}\boldsymbol{-}\boldsymbol{w}_{\boldsymbol{j}}^{\boldsymbol{1}}\left( \boldsymbol{w}^{\boldsymbol{3}}\boldsymbol{\otimes}\boldsymbol{w}^{\boldsymbol{2}} \right)^{\boldsymbol{T}} \right)$.

Figure 5: Derivative of the cost function of ALS optimization step for the penalization $\boldsymbol{L}_{\boldsymbol{0.5}}$ at the interval of interest $\left[ \boldsymbol{0,}\left( \boldsymbol{w}_{\boldsymbol{j}}^{\boldsymbol{1}} \right)_{\boldsymbol{LS}} \right]$ depending on parameter of regularization and least square solution of non-penalized optimization problem (A-C).

[1] Douglas, Ronald G. 1998 Banach Algebra Techniques in Operator Theory, 2nd edition. *New York, New York: Springer-Verlag New York*, Inc. pp. 60–61. ISBN 978-0-387-98377-6.
