# Supplementary figures and images for: Online adaptive group-wise sparse Penalized Recursive Exponentially Weighted N-way Partial Least Square for epidural intracranial BCI
Source: Front Hum Neurosci. 2023 Mar 6;17:1075666. doi: 10.3389/fnhum.2023.1075666 (PMC10025377; doi:10.3389/fnhum.2023.1075666)

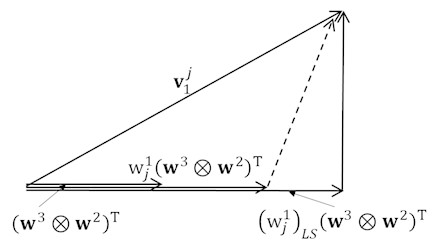

Supplement: Supplementary file 1 [file Data_Sheet_1.zip › Figure 4.JPEG]

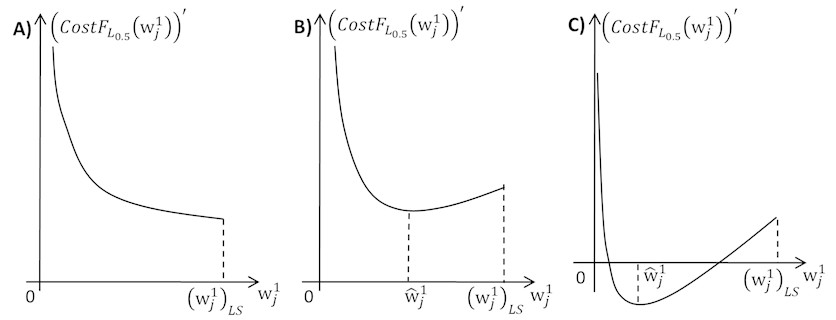

Supplement: Supplementary file 1 [file Data_Sheet_1.zip › Figure 5.JPEG]

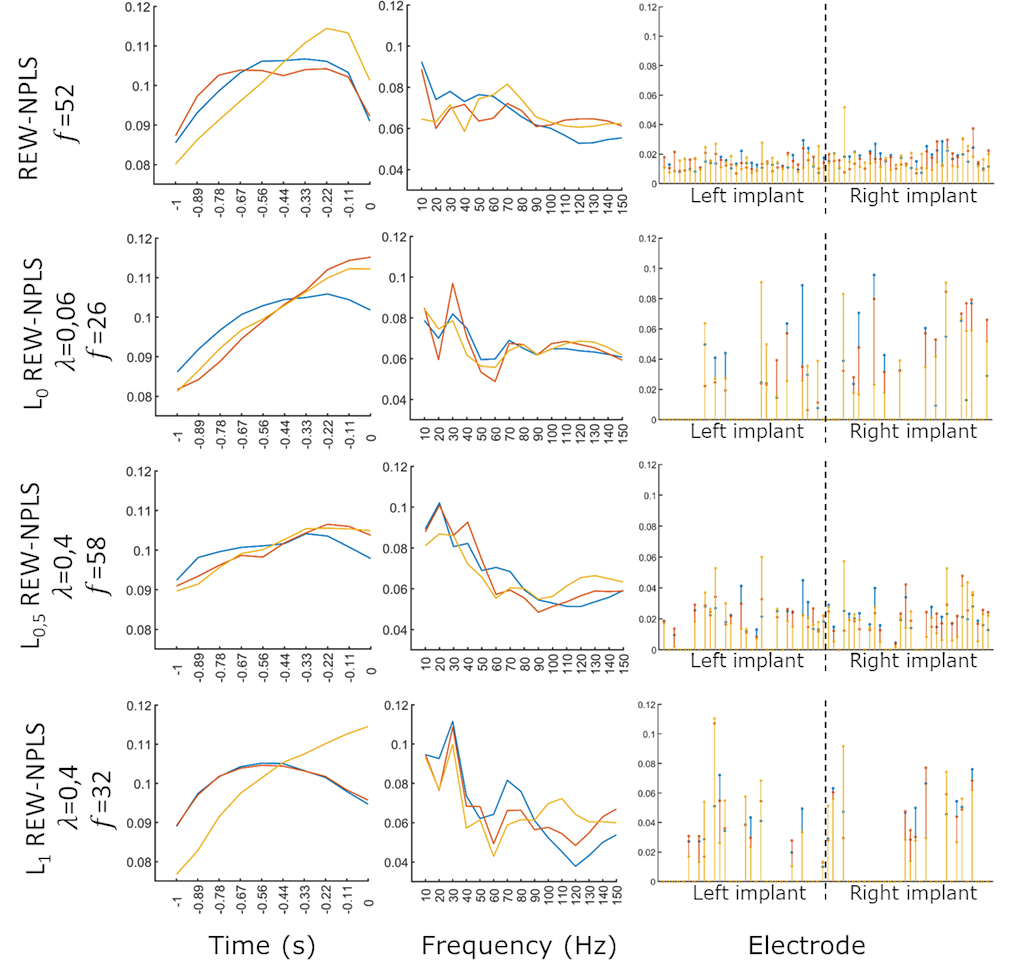

Supplement: Supplementary Figure 1 — Parameter weights of the generic REW-NPLS and Lp, p = 0, 0.5, 1 PREW-NPLS for the left-hand 3D translation. Absolute values of model coefficients are projected into spatial, frequency, or temporal domains. The parameter weights related to the three controlled axes (y1, y2, and y3) are represented using blue, orange, and yellow lines, respectively. [file Image_1.JPEG]

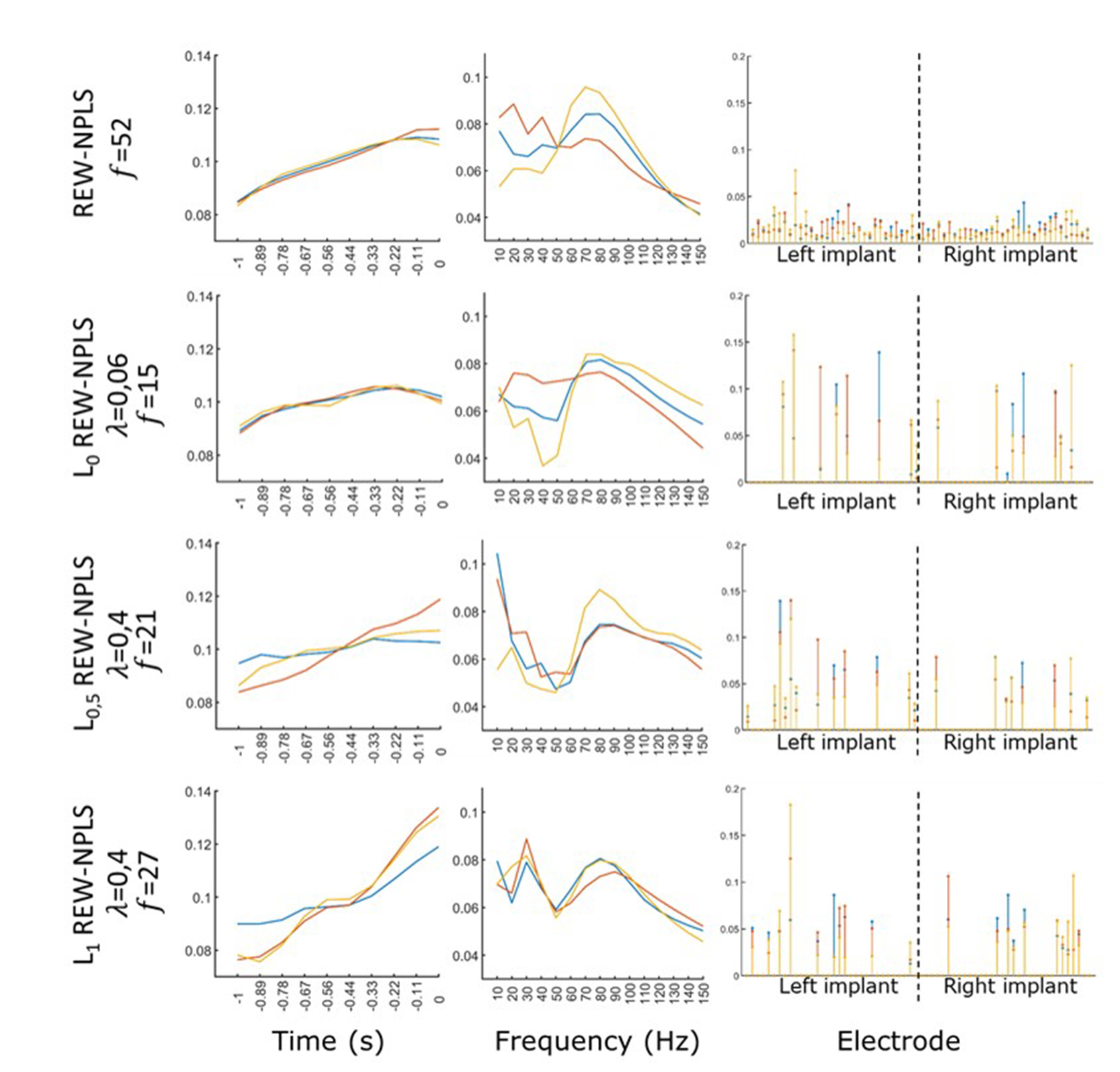

Supplement: Supplementary Figure 2 — Parameter weights of the generic REW-NPLS and Lp, p = 0, 0.5, 1, PREW-NPLS for the right-hand 3D translation. Absolute values of model coefficients are projected into spatial, frequency, or temporal domains. The parameter weights related to the three controlled axes (y1, y2, and y3) are represented using blue, orange, and yellow lines, respectively. [file Image_2.JPEG]

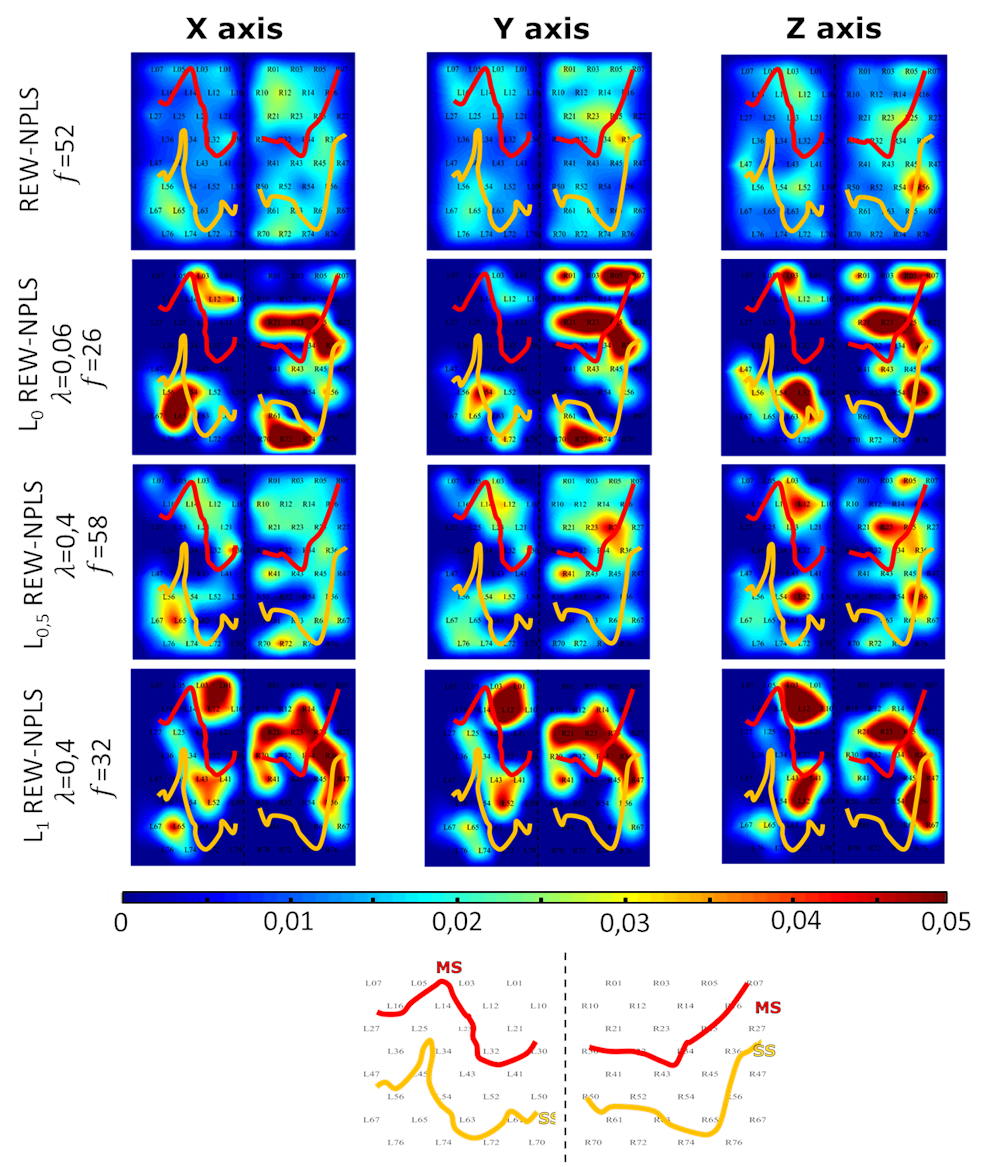

Supplement: Supplementary Figure 3 — Parameter weights of the models identified using generic REW-NPLS and Lp, p = 0, 0.5, 1, PREW-NPLS algorithms (the left-hand 3D translation study) projected into the spatial domain depending on the electrode locations in the implant. The sensory sulcus (SS) and motor sulcus (MS) are shown by yellow and red curves respectively. [file Image_3.JPEG]

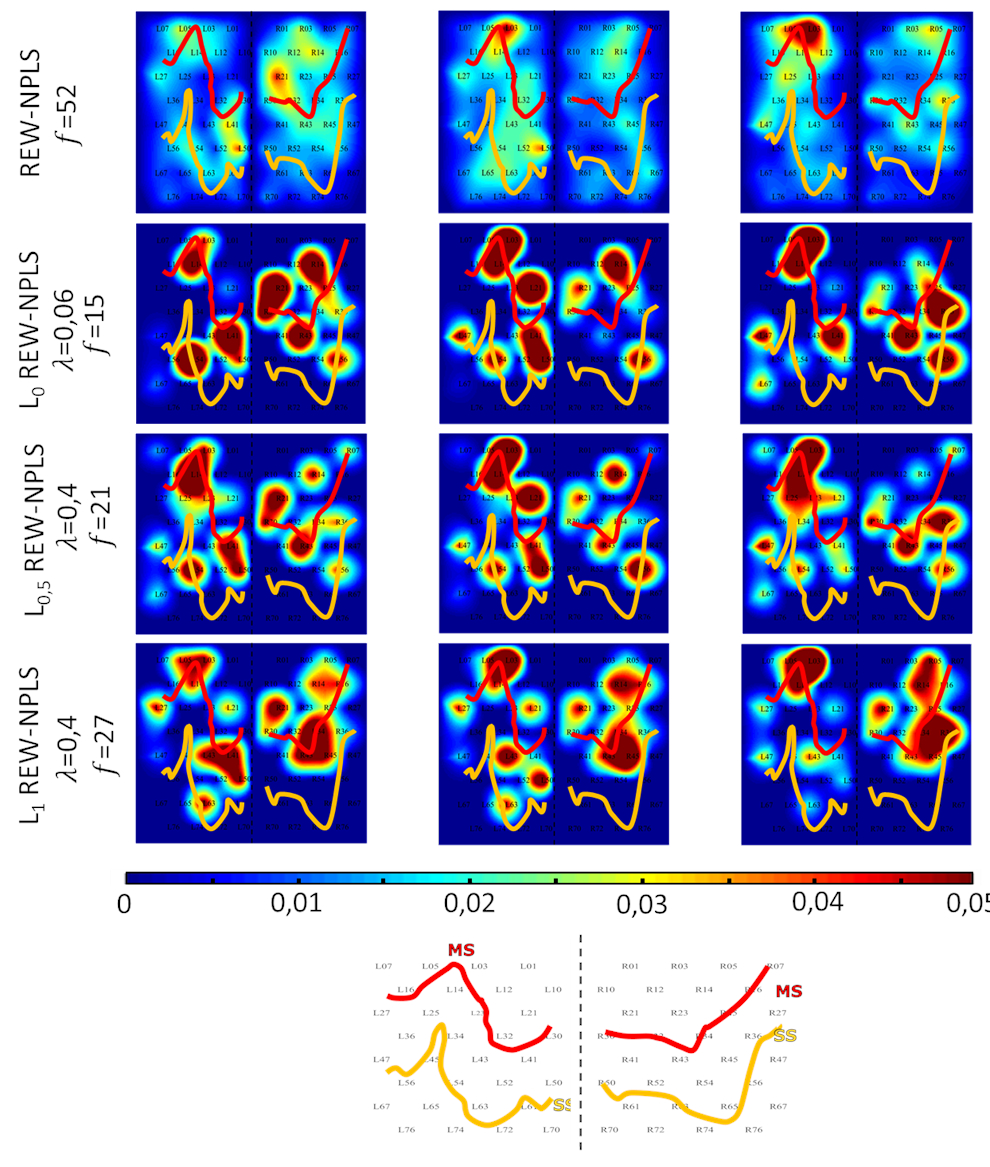

Supplement: Supplementary Figure 4 — Parameter weights of the models identified using generic REW-NPLS and Lp, p = 0, 0.5, 1, PREW-NPLS algorithms (the right-hand 3D translation study) projected into the spatial domain depending on the electrode locations in the implant. The sensory sulcus (SS) and motor sulcus (MS) are shown by yellow and red curves respectively. [file Image_4.JPEG]

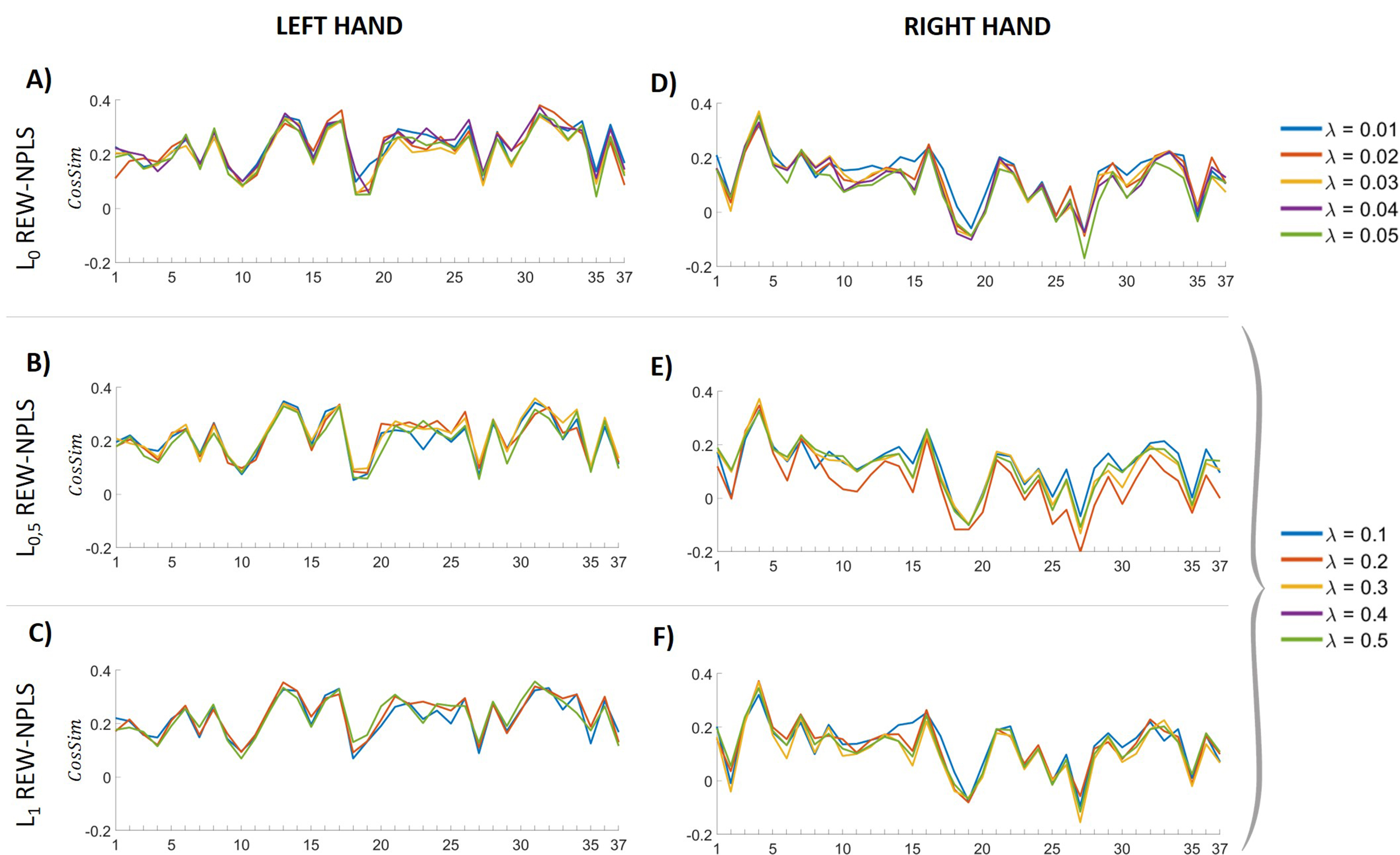

Supplement: Supplementary Figure 5 — The cosine similarity for the Lp penalized REW-NPLS algorithms (p = 0, 0.5, 1), for different λ for 37 test sessions. Cosine similarity of decoding of left-hand 3D translation is depicted in subplots (A–C). Cosine similarity of decoding of right-hand 3D translation is depicted in subplots (D–F). [file Image_5.JPEG]

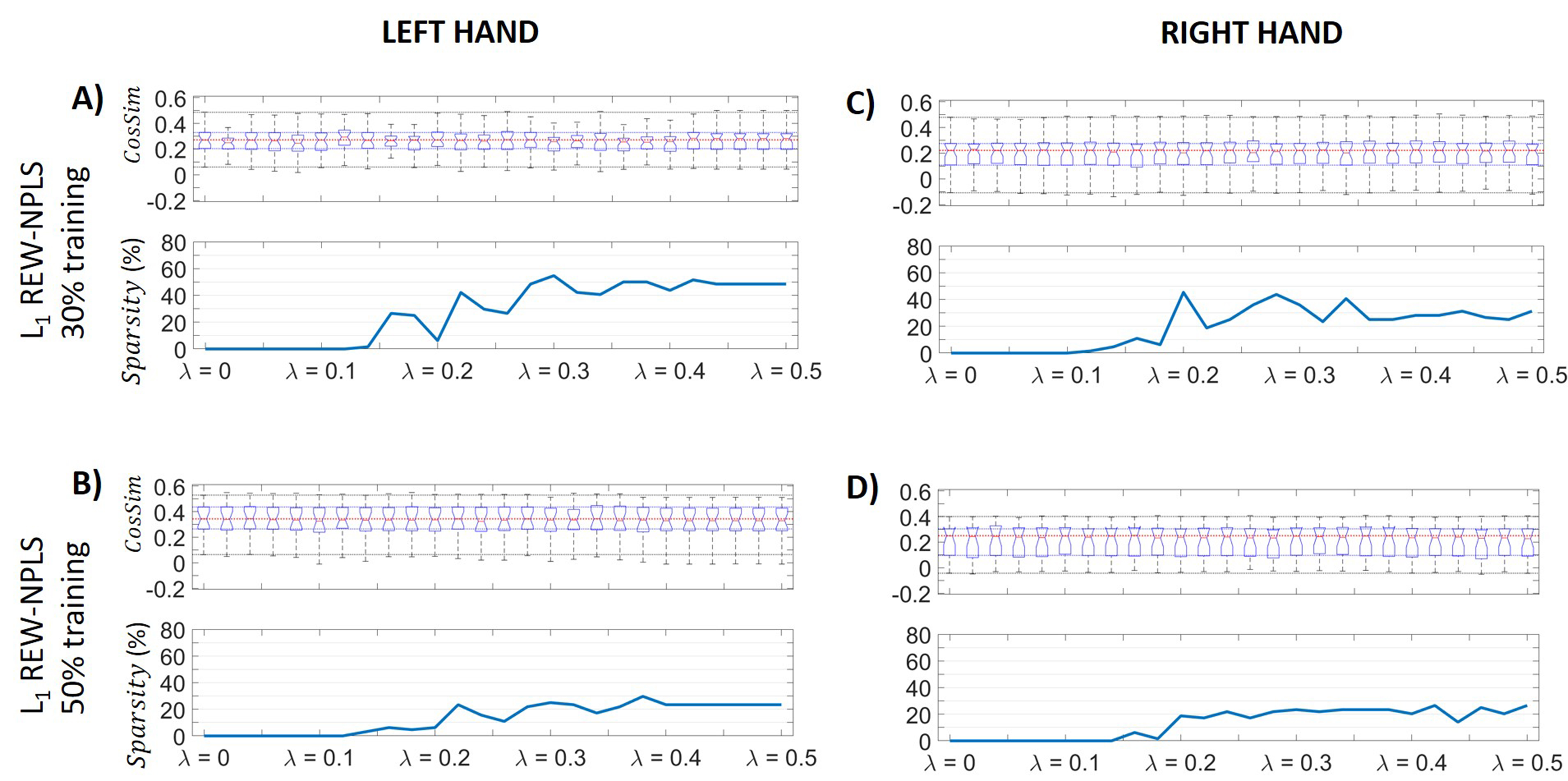

Supplement: Supplementary Figure 6 — The cosine similarity and the model sparsity for the L_1 penalized REW-NPLS algorithm for 3D reaching tasks using 30% (A, C) and 50% (B, D) of the data set for the decoder training. The cosine similarly is summarized using a box plot where the red line is the median the blue lines indicate the 25th and 75th percentiles (Q1 and Q3). Additionally, the whiskers show the upper and lower extreme cosine similarity obtained for the data set. The generic REW-NPLS algorithm results are presented in the first box plot of each sub-plot corresponding to λ = 0. [file Image_6.JPEG]
