# Supplementary material for: Online adaptive group-wise sparse Penalized Recursive Exponentially Weighted N-way Partial Least Square for epidural intracranial BCI
Source: Front Hum Neurosci. 2023 Mar 6;17:1075666. doi: 10.3389/fnhum.2023.1075666 (PMC10025377; doi:10.3389/fnhum.2023.1075666)
Supplement: Supplementary Table 1 — Significance of the performance (CosSimmedian) differences between the REW-NPLS and the L_0, L_0.5, and L_1 PREW-NPLS algorithms for the left hand 3D translation study using the non-parametric paired Wilcoxon signed rank test with and without the Bonferroni correction (α = 0.05 and αBonferroni = 0.00161). [file Table_1.DOCX]

Supplementary Table 1. Significance of the performance ($\boldsymbol{CosSim median)}$ differences between the REW-NPLS and the $\boldsymbol{L}_{\boldsymbol{0}}$, $\boldsymbol{L}_{\boldsymbol{0.5}}$ and $\boldsymbol{L}_{\boldsymbol{1}}$ PREW-NPLS algorithms for the left hand 3D translation study using the non-parametric paired Wilcoxon signed rank test with and without the Bonferroni correction ($\boldsymbol{\alpha=0.05}$and $\boldsymbol{\alpha}_{\boldsymbol{Bonferroni}}\boldsymbol{=0.00161}$). The bold P-values show statistically significant differences with $\boldsymbol{\alpha=0.05}$, the bold underlined P-values show statistically significant differences for the test with the Bonferroni correction.

| $\lambda$ | $0.02$ | $0.04$ | $0.06$ | $0.08$ | $0.10$ | $0.12$ | $0.14$ | $0.16$ | $0.18$ | $0.2$ | $0.22$ | $0.24$ | $0.26$ | $0.28$ | $0.30$ | $0.32$ | $0.34$ | $0.36$ | $0.38$ | $0.40$ |
| --- | --- | --- | --- | --- | --- | --- | --- | --- | --- | --- | --- | --- | --- | --- | --- | --- | --- | --- | --- | --- |
| $L_{1}$ | $0.73$ | $0.15$ | $\boldsymbol{0}.\boldsymbol{02}\boldsymbol{1}$ | $\boldsymbol{0}.\boldsymbol{018}$ | $0.53$ | $\boldsymbol{0}.\boldsymbol{0016}$ | $\boldsymbol{0}.\boldsymbol{0005}$ | $0.24$ | $\boldsymbol{0}.\boldsymbol{0012}$ | $0.5$ | $0.15$ | $\boldsymbol{0}.\boldsymbol{027}$ | $0.24$ | $\boldsymbol{0}.\boldsymbol{0023}$ | $0.24$ | $0.21$ | $0.24$ | $0.21$ | $0.21$ | $0.21$ |
| $L_{0.5}$ | $0.21$ | $0.84$ | $0.39$ | $0.99$ | $0.21$ | $\boldsymbol{0}.\boldsymbol{025}$ | $0.07$ | $0.06$ | $0.47$ | $0.96$ | $\boldsymbol{0}.\boldsymbol{0003}$ | $0.59$ | $0.83$ | $0.18$ | $\boldsymbol{0}.\boldsymbol{007}$ | $\boldsymbol{0}.\boldsymbol{0005}$ | $\boldsymbol{0}.\boldsymbol{0005}$ | $\boldsymbol{0}.\boldsymbol{0005}$ | $\boldsymbol{0}.\boldsymbol{0005}$ | $\boldsymbol{0}.\boldsymbol{0005}$ |

| $\lambda$ | $0.002$ | $0.004$ | $0.006$ | $0.008$ | $0.010$ | $0.012$ | $0.014$ | $0.016$ | $00.18$ | $0.02$ | $0.022$ | $0.024$ | $0.026$ | $0.028$ | $0.030$ | $0.032$ | $0.034$ | $0.036$ | $0.038$ | $0.040$ |
| --- | --- | --- | --- | --- | --- | --- | --- | --- | --- | --- | --- | --- | --- | --- | --- | --- | --- | --- | --- | --- |
| $L_{0}$ | $0.22$ | $\boldsymbol{10}^{-\boldsymbol{4}}$ | $\boldsymbol{0}.\boldsymbol{012}$ | $0.45$ | $\boldsymbol{0}.\boldsymbol{0006}$ | $0.34$ | $\boldsymbol{0}.\boldsymbol{01}$ | $0.11$ | $\boldsymbol{0}.\boldsymbol{009}$ | $0.40$ | $\boldsymbol{10}^{-\boldsymbol{5}}$ | $0.10$ | $\boldsymbol{0}.\boldsymbol{04}$ | $\boldsymbol{0}.\boldsymbol{0017}$ | $0.13$ | $0.47$ | $\boldsymbol{0}.\boldsymbol{012}$ | $\boldsymbol{0}.\boldsymbol{044}$ | $\boldsymbol{0}.\boldsymbol{013}$ | $\boldsymbol{0}.\boldsymbol{008}$ |

| $\lambda$ | $0.042$ | $0.044$ | $0.46$ | $0.48$ | $0.50$ |
| --- | --- | --- | --- | --- | --- |
| $L_{0}$ | $\boldsymbol{0}.\boldsymbol{008}$ | $\boldsymbol{0}.\boldsymbol{008}$ | $0.67$ | $0.67$ | $0.67$ |
